# Evaluation of the Phytochemistry–Therapeutic Activity Relationship for Grape Seeds Oil

**DOI:** 10.3390/life13010178

**Published:** 2023-01-08

**Authors:** Manuel Alexandru Gitea, Simona Gabriela Bungau, Daniela Gitea, Bianca Manuela Pasca, Anamaria Lavinia Purza, Andrei-Flavius Radu

**Affiliations:** 1 Department of Agriculture, Horticulture, Faculty of Environmental Protection, University of Oradea, 410048 Oradea, Romania; 2 Department of Pharmacy, Faculty of Medicine and Pharmacy, University of Oradea, 410028 Oradea, Romania; 3 Doctoral School of Biomedical Sciences, University of Oradea, 410087 Oradea, Romania

**Keywords:** grape seeds oil, *Vitis vinifera* L., phytochemistry, bioactive compounds, biologic activity, extraction techniques, therapeutic activity

## Abstract

Seeds’ abundant biologically active compounds make them a suitable primary platform for the production of natural extracts, innovative foods, medicines, and cosmetics. High levels of industrial and agricultural residues and byproducts are generated during the processing of grapes, although some parts can also be repurposed. This paper examines the phytochemical composition, manufacturing processes, and health-improving attributes of many varieties of grape oil derived using various extraction methods. Since the results are influenced by a range of factors, they are expressed differently among studies, and the researchers employ a variety of measuring units, making it difficult to convey the results. The primary topics covered in most papers are grape seed oil’s lipophilic fatty acids, tocopherols, and phytosterols. In addition, new methods for extracting grape seed oil should therefore be designed; these methods must be affordable, energy-efficient, and environmentally friendly in order to increase the oil’s quality by extracting bioactive components and thereby increasing its biological activity in order to become part of the overall management of multiple diseases.

## 1. Introduction

Large quantities of significant agricultural and industrial wastes and byproducts are produced during the processing of grapes, and these materials may be used again in a variety of ways. The amount of solid byproducts produced during processing has been calculated to be greater than 0.3 kg for every kg of crushed grape fruit. In the various industrial processes of grape berries, seeds make up most of the weight of the processed grapes, 7–20%, and can be found in pomace, for example. Even though the skins and vascular fruit tissues are also taken from the pomace together with the grape seeds, they can still be removed easily via technological separation and sifting. Seeds contain high levels of bioactive antioxidants and can be utilized as a source of raw materials to create natural extracts, new foods, cosmetics, and pharmaceuticals. As a result, the manufacturing of grape seed oil (GSO) improves waste management, which could boost the financial performance and sustainability of the primary industrial process [1].

**Figure 1:**
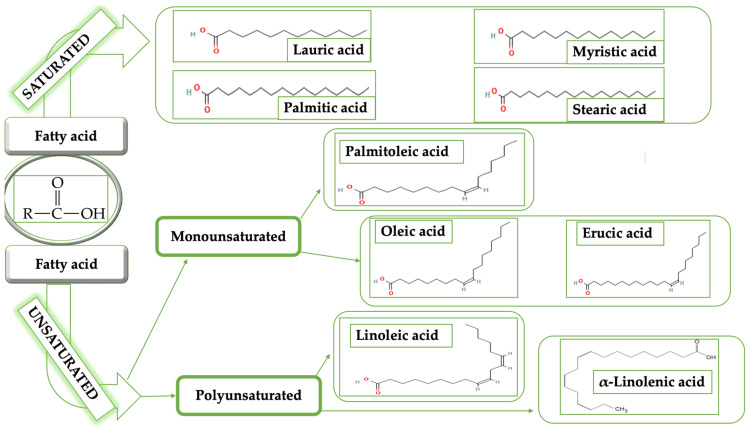
General classification of predominant fatty acids detected in GSO.

Grape seeds are used to obtain vegetable oil. Vegetable oils are still successfully applied in the cosmetic and pharmaceutical industries today due to their therapeutic and cosmetic benefits on skin health, some of which have been known for ages. For instance, they serve as excipients, active compounds, and extraction agents. According to its chemical composition, GSO is mostly made up of triglycerides (usually 99%) and unsaponifiable materials (typically 1%). Fatty acids (FAs) and glycerol are esterified to form triglycerides [2]. FAs are divided into saturated, mono-, and polyunsaturated groups based according to how many double bonds they possess, which determines how susceptible they are to challenges brought on by light, heat, or oxygen [3]. In Figure 1 are categorized highly prevalent FAs identified in GSO.

Carotenoids, phytosterols, squalene, phenols, and vitamin E are the major unsaponifiable constituents. Vegetable oils of the finest quality are often extracted using CO_2_ extraction and cold pressing, without further refining, as they are not subjected to changes caused by temperature or oxidation and do not include solvent residuals. Recently, other methods of obtaining essential oils have been described in the literature (ultrasound-assisted extraction, microwave-assisted extraction, supercritical fluid extraction, and gas-assisted mechanical expression) [4,5,6].

Vegetable oils’ cutaneous effects are dependent on unsaponifiable matter, triglycerides, and FAs. There is still a lack of scientific data on the precise mechanisms involved and the range of cutaneous effects. Even so, significant strides have been achieved recently in the field of clinical research, and mounting data support the scientifically-based use of vegetable oils in domains including cosmetic science, medicine, and pharmacy [2].

Since 1929, it has been known that specific fats play a crucial role in skin structure, when Burr and Burr first described a syndrome brought on by strict fat reduction in diet (which mainly manifested as cutaneous symptoms, including hair loss, increased water loss, and erythema with scaling and itching) [7,8]. These fats are best described as “necessary” because the human body cannot produce them; therefore, consumption is the only way to obtain them. Linoleic acid (LA) and α-linolenic acid (ALA), two polyunsaturated FAs (PUFAs), were the inducting acids for the sequence of elongation to very long-chain PUFAs (more than 22 C-atoms) were described as “essential fatty acids”. Alpha linolenic acid is considered essential because it cannot be synthesized by the body; the main source of obtaining it is food. ALA is found in high concentrations in plant seeds. Omega-3(ω-3) and omega-6(ω-6) are the two groups into which PUFAs are separated. While ω-6 acids have it in the omega six-position, which is the sixth bond from the methyl end of the fatty acid, accordingly, ω-3 fatty acids share a terminal carbon-carbon double bond in the omega three position, which is the third bond from the methyl end of the acid. ALA is categorized as a ω-3 PUFA, while LA belongs to the ω-6 family. These FAs always have cis-configuration double bonds, which indicates that two hydrogen atoms are located on the same side of the double bond [9].

Omega-3 FAs are primarily recognized for their beneficial effect on cardiovascular conditions, but extensive research suggests that these acids play an important role in other pathologies as well. LA is the main omega-6 in food, it is also considered an essential fatty acid because it cannot be synthesized in the body. Unsaturated FAs are abundant in GSO, particularly LA, which is a sizeable component of the seed [10]. 

In the human body, omega-6 FAs, especially LA, are converted to arachidonic acid, which is incorporated into cell membranes. Since arachidonic acid is involved in the early stages of inflammation, there has been much debate about the harmful effects of omega-6 FAs, due to the fact that they promote inflammation. However, LA also generates anti-inflammatory molecules. Thus, the vascular endothelium level, omega-6 FAs have anti-inflammatory properties, suppressing the production of adhesion molecules, chemokines, and interleukins, which are key mediators of the atherosclerosis process [11]. 

The beneficial outcomes of PUFAs may be favored by a number of different processes, including changes in the lipid composition of cell membranes, gene expression, signal transduction, and cellular metabolism [12]. The metabolic processes in the human body are, nevertheless, antagonistically impacted by ω-3 and ω-6 FAs. PUFAs are currently the preferred ingredients of “specialty oils,” which are employed as cosmeceuticals or nutraceuticals because they have unique nutritional and functional qualities. PUFAs, particularly ω-3 PUFAs, are highly valued for the health system because of their possible uses in prophylactic treatment, as well as in intervention for the majority of prevalent inflammatory conditions, including inflammatory skin diseases such as psoriasis, atopic dermatitis (AD), and acne. This is due to a deeper comprehension of their physiological and functional properties as well as their advantages for health. Health professionals currently have access to effective features such as nutrilipomics and lipidomics that direct them to offer the most appropriate and individualized FA nutritional supplements for the therapies of their patients, as well as the disease prophylaxis in a wide range of clinical domains, including the sector of dermatology [13].

The majority of cooking-related seed and vegetable oils, such as grape seed, poppy seed, sunflower, safflower, wheat germ, corn, palm, soybean, hemp, cottonseed, and rapeseed, are important sources of ω-6 PUFAs in the form of LA with low levels of ω-3 FAs, alpha-linolenic acid (ALA) being the first indicated [14]. When compared to ω-6 Fas, ω-3 Fas are typically insufficiently consumed due to their scarce sources. Flaxseed, soybean, canola, walnuts, and green leafy vegetables all contain ALA [15]. 

Vegetable oils’ triglyceride content has been extensively studied, while investigations on unsaponifiable molecules are rare. Flavonoids, phytosterols, phenolic acids, tocotrienols, tocopherols, and carotenoids, which are isolated unsaponifiable chemicals, were shown to have anti-inflammatory, anti-acne, and anti-dermatitis properties. They also have moisturizing, regenerative, anti-wrinkle, and photoprotective effects. Although they are essential structural elements of vegetable oils, unsaponifiable chemicals’ effects on the skin have not been extensively studied in clinical investigations [2]. Similarly to other plant-based foods, the antioxidant activity of grape seed oil is closely correlated with the amount of vitamin E [16,17], the concentration of active compounds from fruits and seeds, respectively from other parts of the plant, varying according to numerous factors, as follows: the cultivation area, the rheological properties of the soil, the climatic characteristics, the stage of maturation/ripening of different parts of the plant, the method of harvesting/preserving plant material until the determinations/analysis, etc. [18].

Additionally, diets high in particular FAs may help avoid a number of illnesses or conditions. Consumer interest in enhancing their diet is currently rising. Vegetable oils with distinctive fatty acid profiles and other beneficial components, such as natural antioxidants and phytosterols, have become increasingly popular due to these characteristics and other advantages [19]. 

Recently, pharmaceutical, and economic trends have focused on the search for new drugs in the field of plant secondary metabolites as substances for the management of different pathologies. Due to its important biologically active components, GSO can be used for medical purposes. 

The present paper offers, in a novel approach, a comprehensive presentation of GSO obtained by different physicochemical methods and having several recognized traditional uses, focusing on the correlation between bioactive compounds identified by experimental studies and biological activity showing potential adjuvant use in various disorders. Moreover, another contribution to the evaluated topic is the creation of a scientific framework by updating the state of knowledge in the field. 

## 2. Methodology of Research

The present article evaluates and centralizes scientific publications on the comprehensive description of GSO, through an advanced search of the available literature. In this regard, publications concerning the botanical characteristics of *Vitis vinifera* L., methods of obtaining GSO with the main advantages and limitations, a comprehensive description of the phytochemical composition, traditional uses, focusing on the biological activity of phytocompounds that are exploited through multiple effects on various pathologies and that present a significant relevance based on clinical and experimental studies, as well as history in pharmaceutical uses were targeted according to the methodology. 

**Figure 2:**
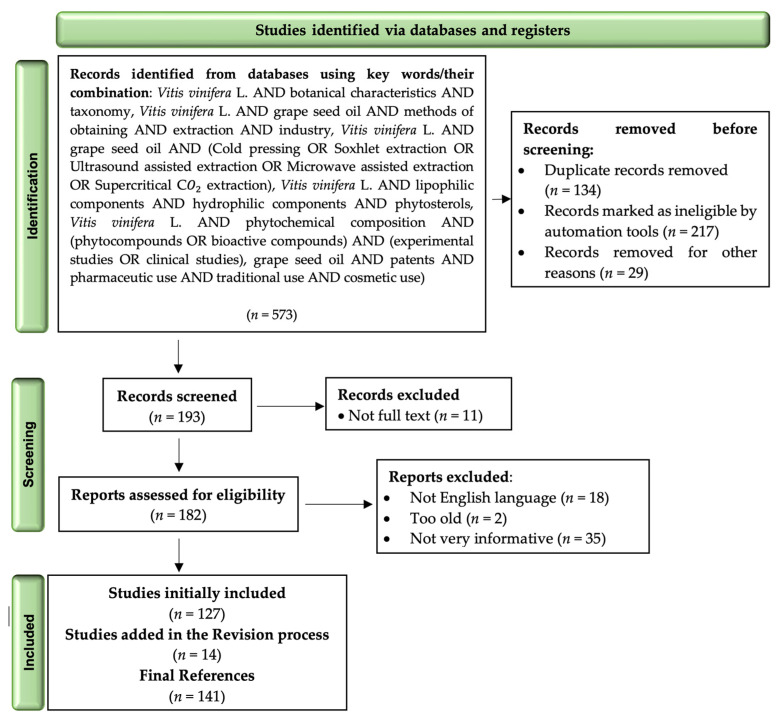
Literature selection depicted in a PRISMA 2020 flow diagram.

The medical literature search involved the use of large and valuable databases providing numerous biomedical results (e.g., PubMed, Nature, Springer Link, and ScienceDirect). The detailed information search flows underlying the methodology research using predefined algorithms, and applying Boolean search operators, is described in the PRISMA-based operating processes, together with examples of the selection, evaluation, and final inclusion criteria (Figure 2), as recommended by Page et al., 2021 [20]. 

Following the screening, papers that were not published in English, were not very comprehensive, or were not article-type publications were excluded. To validate the medical information in the present research, a total of 141 bibliographic references were examined and mentioned.

## 3. Botanical and Taxonomical Description of *Vitis vinifera* L.

*Vitis vinifera* L. is a creeping species that grows 12–15 m tall. The taproot reaches a depth of 2 to 5 m and sometimes up to 12–15 m or even more. *Vitis vinifera* L. has flaky bark and the stems, grow through their tip. During their hardening, the stems become woody branches that can reach a great length. A branch consists of several internodes separated by nodes, on which grow leaves, flowers, and tendrils. Leaves are alternate, palmately lobed, with three to five pointed lobes, glossy dark green above, light green below. The flowers are small and greenish to white, clustered in inflorescences [21,22]. The calyx is monosepalous with five short teeth. The corolla consists of five petals, united at the base. The five stamens are provided with glands. The ovary is superior and bears a very short style with a button-shaped stigma. The fruits have different shapes depending on the subspecies. In general, the fruit is a berry, known as a grape, ovoid or globose in shape, dark blue or greenish in color, and up to 3 cm in diameter. The color of unripe fruit is usually green and ripe fruit is dark purple. Ripe fruits are grey. The shape of the seed was pear-shaped, the color of the seed was dark brown, the surface is smooth with ridges on the back surface, the apex is discoidal, the size is 4–8 mm long, and the taste is bitter [22]. 

**Figure 3:**
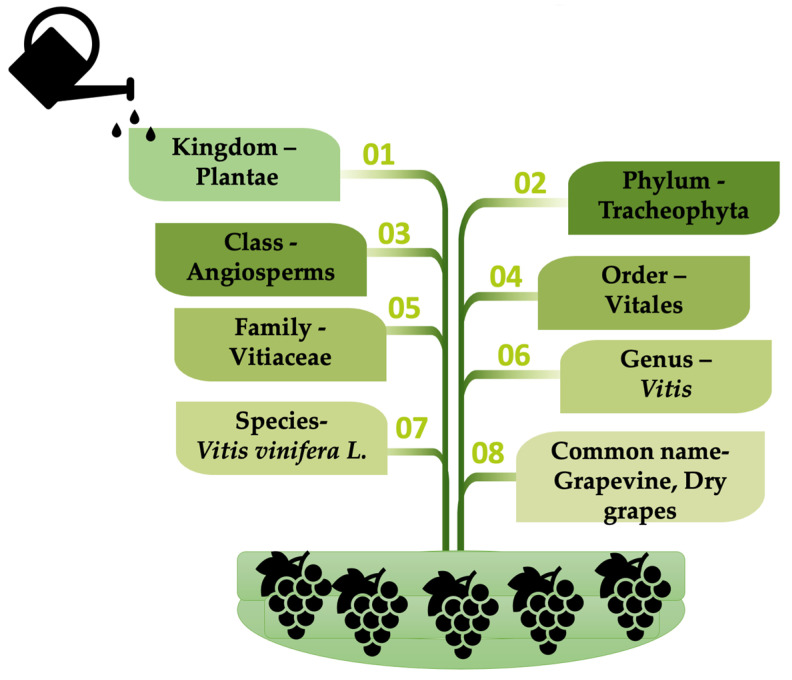
Taxonomical classification of *Vitis vinifera* L.

The scientific classification of *Vitis vinifera* L. is displayed in Figure 3.

## 4. Strategies to Extract Oil from Grape Seeds

In order to increase the overall production, oil from oil seeds is typically extracted on an industrial scale by screw pressing, which is frequently followed by procedures involving organic solvent extraction. However, in order to enhance oil extraction output from seeds with little oil concentration, such as grape seeds, solvent extraction is preferred. These techniques are used to extract grape stem crude oil, which needs to be refined before it can be utilized in the grocery industry. Through the physico-chemical procedures of purification (decolorization, degumming, deodorization, and deacidification), several components that give the oil its distinctive color and fragrance are eliminated [23].

Although this technique produces significant oil yields, it has a number of drawbacks, including environmental pollution, non-selective solubility towards lipid-soluble substances, etc. [24].

Several modern extraction techniques minimize the detrimental effects of heat degradation and satisfy the requirements for “green” extraction methods. In addition, they decrease or completely avoid the utilization of organic solvents, provide energy savings, speed up processing, improve mass transfer, lower processing temperatures, and boost extraction outcomes with high-quality extracts. Alternative strategies for enhancing the antioxidants extracted from grape seeds include extractions assisted by microwave, ultrasound, or high-pressure processing [1]. 

Alternative oil extraction technologies primarily concentrate on water processes (enzymatic) and supercritical fluid extraction in addition to solvent and mechanical (hydraulic pressing or screw pressing) procedures. Utilizing supercritical CO_2_ to study the supercritical fluid extraction with various source ingredients (e.g., rapeseed, linseed, grape seeds). Processing conditions, such as temperature and CO_2_ pressure, can be used to boost oil production. However, due to a growth in CO_2_’s solvent power, which results in higher operating costs, intense pressure must be used to obtain high oil production [23,24]. Gas-Assisted Mechanical Expression (GAME) is a different expression method that was created with the goal of reducing energy and costs. The fundamental idea behind GAME is that while pressing, oil is partially displaced with CO_2_, increasing the amount of oil that is produced. Seed preparation in supercritical CO_2_ is succeeded by oil expression in uniaxial compression when investigations of this industrial procedure are performed. Another advancement in industrializing this method would be the utilization of a constant stream of CO_2_ during extraction [23].

**Figure 4:**
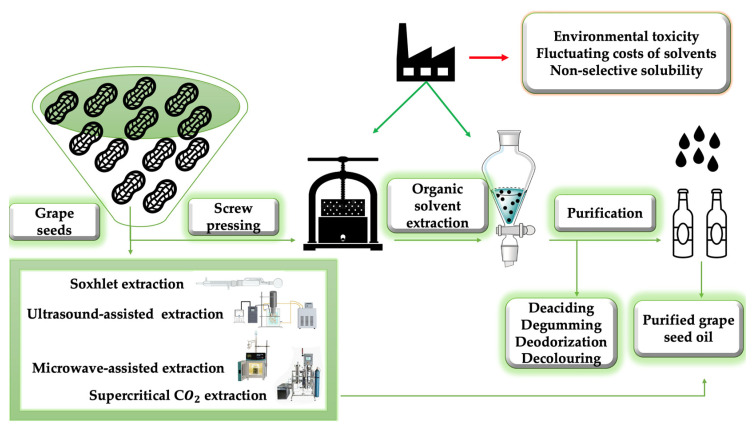
Methods of obtaining grape seed oil.

After the grapes have been harvested and squeezed to make wine, the stems are removed, dried, and squashed from the resulting marc. Figure 4 illustrates the most common methods of oil extraction.

### 4.1. Cold Pressing

Some consumers prefer natural, nutritive, and safe dietary supplements to maintain overall wellbeing and illness prevention, such as cold-pressed seed oils, due to their considerable chemical properties and health-promoting qualities. Cold pressing is easily performed, environmentally sustainable, and uses a reduced amount of energy. When considering the equipment expenditure cost and the cost of the finished product, the cold-press technique is shown to be cheaper and less labor-intensive than other extraction processes. This is particularly noticeable when compared to supercritical carbon dioxide (CO_2_) extraction. As a result, cold pressing can produce high-quality oils at better prices, and it can be employed in industrial manufacturing [25]. 

Based on extraction methods, there are variances in the final volumes of oil. Compared to hot pressing, supercritical carbon dioxide (CO_2_) extraction, and solvent extraction, the volume of oil obtained through cold pressing is lower. Since cold-pressed oils are organic, nutritive, and secure food products, customer preference for using them has grown recently. Due to the absence of heat treatment or any organic solvent in the extraction process, cold-pressed seed oils contain significant chemical qualities, a high nutritional value, unique sensory characteristics, and health-promoting elements. The cold-pressing method is, therefore, more effective in preserving oil antioxidant compounds and advantageous phytochemicals such FAs, sterols, tocopherols, and antioxidant phenolic compounds [26]. 

The impact of screw pressing factors on the quality of the oil (peroxide level, free FAs content, oxidative stability) is hardly studied. Yet, throughout the procedure, quite high temperatures of 60–68 °C can be attained, which may influence the content and quality of the oil. A 12-experiments Taguchi experimental model was used to evaluate the study’s primary material and manufacturing parameters by Natacha Rombaut et al. Variables included the grape seed variety, screw rotation speed (40 and 70 rpm), preheating temperature (90 and 120 °C), and die diameter (10 and 15 mm). The factor that had the greatest impact on the answers within the study was the variety of grape seeds. The only factors influencing the oil yield were die diameter and screw rotation speed [25]. 

### 4.2. Soxhlet Extraction

Although solvent extraction generates a better yield, it comes with drawbacks of the presence of hazardous residues in the finished product, longer production process, and reduced nutritive value of the oil. Oils cold extraction is frequently associated with a lower ultimate yield [27]. 

N-hexane is frequently used in Soxhlet extraction of grape oils because it is non-selective and concurrently eliminates waxes and non-volatile pigments. As a result, the extracted materials are darkish, thick, and tainted with noxious solvent residue [1]. This method is mostly used in research laboratories and less often in the industry. As extraction solvents, the most used are n-hexane, petroleum ether, the extraction time being 6 h, at the solvent’s boiling point (60 to 70 °C). A rotary evaporator was used to recover the solvent, with the bath temperature set at 40 °C and the rotation speed set at 30 rpm [27]. 

### 4.3. Ultrasound-Assisted Extraction

Acoustic cavitation is the foundation of ultrasound-assisted extraction (UAE). Bubbles are formed during the ultrasonic treatment negative pressure phase, which is when the pressure and temperature spikes and subsequent collapse of the bubbles first occur. The ensuing “shock waves” shatter the cellular walls and allow the solvent to enter the plant components, increasing the yields of the extraction [1]. 

Lately, it was shown that, despite taking less time to extract the oil, the amount of GSO produced by ultrasound separation was comparable to that achieved using the Soxhlet method [27,28]. Rita de Cássia de Souza’s studies suggest that performing ultrasound pretreatment could be an effective way to increase separation efficiency and extract phenolic compounds in all extraction techniques, particularly supercritical fluid extraction, which, in addition to being highly efficient, does not require the use of organic solvents [27]. 

Generally, the extraction conditions through UAE are a temperature of 50 °C, extraction time of 40 min, and sonication power of 60 W/L, this sonication power can be modified. The extraction solvent was then vacuum-evaporated at 40 degrees Celsius while the extracts were filtered [1]. 

### 4.4. Microwave-Assisted Extraction

Microwaves, which are nonionizing electromagnetic waves with a range between 300 MHz and 300 GHz, are used in microwave-assisted extraction (MAE). Thus, electromagnetic waves are converted into thermal energy, causing the matrix to heat up both internally and externally without a temperature gradient. If enough thermal energy is produced, this targeted heating destroys the plant matrix’s cell walls and results in the release of the target substances into the extraction medium [1]. For microwave-assisted extraction, ground grape seed samples are used, mixed with a solvent, usually n-hexane [29]. Through a hole in the top of the casing, samples are inserted in the microwave extractor with an associated condenser. The mixture of grape seed powder and solvent and even the sample radiation power (300 W–600 W) differ from one researcher to another. Additionally, the wave irradiation can be continuous for a longer period of time, of several minutes, or for short periods of a few seconds, followed by an irradiation break of a few seconds [30]. 

According to data, MAE was effective in retrieving tocopherol-rich samples, whose combined level for red and white grapes was their total physiologically active material with antioxidant characteristics [1]. 

### 4.5. Supercritical Fluid Extraction

Due to the improved quality of the resulting products and the fact that it is regarded as a clean technology (ecofriendly/green process), supercritical fluid extraction (SFE) has gained significant interest in recent years as a possible alternative to conventional extraction, particularly in the pharmaceutical, cosmetic, food, and nutraceutical industries [27]. A better quality of the product that is comparable to mechanical pressing is another attribute of GSOs collected by SFE [1]. 

When analyzing the extracts produced by SFE in contrast to those produced by other traditional extraction techniques, it is found that the number of chemicals produced by SFE using the same matrix is significantly larger, according to Machado et al. Although there are more extracted molecules, the extraction procedure frequently has lower productivity, which might suggest greater selectivity. Recently, the use of coupled ultrasound in SFE has been suggested as a technique to speed up the procedure, improve yield, and extract more valuable chemicals [27]. 

#### Supercritical CO_2_ Extraction

SFE is superior to traditional methods, permitting the use of environmentally friendly production solvents, typically employing supercritical carbon dioxide (CO_2_) as the solvent/extraction fluid, and is perfect for the separation of thermally sensitive chemicals because low temperatures may be used in the procedure [27]. 

Supercritical CO_2_ extraction exhibits the attributes that are similar to both gases and liquids, such as diffusivity, viscosity, and density. CO_2_ has a critical pressure of 73 bars and a melting point of 31 °C, making it a sustainable, inexpensive, non-toxic, and inflammable solvent. Due to the fact that it can be recycled during processing, it can lower industrial energy expenditures overall. Additionally, because supercritical CO_2_ can be removed entirely by a pressure decrease, fluid residues are not left in the finished product. For polar phytocompounds located within the cell wall, co-solvents and modifiers, including acetone, ethanol, and methanol, may be incorporated to enhance their solubility [31]. Additionally, by altering the operating conditions, including pressure and temperature, supercritical CO_2_ provides selectivity in the extraction of specific target molecules, and the scientific publications already contain an approximative cost estimate of commercial SFE scale-up from the laboratory [1].

Throughout comparing the extraction with n-hexane, Hatem Ben Mohamed et al.’s statistical research revealed that supercritical CO_2_ extraction of GSO allowed for higher overall tocols (362–567 mg/kg) and carotenoids (2.7–4.8 mg/kg) levels, which were linked to greater lipophilic antioxidant activity (4.9–8.1 mol trolox/g oil) [32]. 

According to the literature, ultra-critical CO_2_-extracted oils had higher lipophilic antioxidant values than hydrophilic antioxidant values, which were unaltered by the extraction process. This indicated that, in order to acquire the appropriate antioxidant potential, the kind of extraction and the accompanying parameters should be carefully examined for the extraction of oil from grape seeds [1]. 

For examining the effectiveness of three different oil extraction processes for obtaining an oil rich in polyphenols, Natacha Rombaut et al. compared screw pressing, gas-assisted mechanical expression, and supercritical CO_2_ percolation extraction. Although supercritical CO_2_ procedure enables a higher amount of polyphenol in co-extraction with oil, screw pressing is the most effective method for obtaining a greater amount of GSO [23]. 

Jakobović, M. et al., followed the influence of the drying process of grape seeds in obtaining oil by supercritical CO_2_ extraction. The findings of the research indicate that drying the seeds in a dryer is necessary to lower the humidity level of the seeds, which has a knock-on effect on the oil. As the excessive moisture content negatively impacts oil quality, this will help to improve oil properties. Applying green technology, such as SFE, maximizes the oil characteristics [33]. 

According to Xin Wen et al., the Cabernet Sauvignon seed oil with the highest concentrations of total sterols, squalene, total phenolics, and total tocopherols was extracted using supercritical carbon dioxide extraction [34]. 

F. Agostini et al. observed that the varying yields of oil may be caused by a number of factors, including temperature, pressure, extraction time, and the type of grape utilized in various experiments. As a result of the supercritical fluid increased density, the solubility of seed oil typically increases with increasing pressure. Additionally, rising temperature raises the vapor pressure of the solute and lowers the density of the supercritical fluid, increasing the amount of oil that can be extracted. The Bordo variety is shown to have significant quantities of procyanidins, total phenolics, FAs, α-Tocopherol, and total phenolics, according to the data from the current study [35]. 

Rita de Cássia de Souza et al. suggested that the process of ultrasound pretreatment would be a practical way to increase extraction efficiency and get phenolic compounds in all extraction procedures, particularly by SFE, which, in addition to its high efficiency, does not require the usage of organic solvents. Additionally, for the extraction of α-tocopherol, either with or without ultrasound pretreatment, the measured quantity increased [27].

## 5. Phytochemical Composition of Grape Seeds

Most of the studies carried out so far are based on the extraction of the active principles from the grape skin or on the characterization of the aqueous or alcoholic extracts of grape seeds. Since they are abundant in phenolic compounds, grape seeds may be good for human health [36]. Consequently, it has been suggested that GSO is a viable dietary supplement that could prevent or treat physiological abnormalities linked to long-term illnesses [37]. Superoxide radical scavenger properties have been found in grape seeds [38]. 

Due to its high concentration of hydrophilic components, such as phenolic compounds and lipophilic components, GSO has become more noticeable as a dermatological product [39]. 

### 5.1. The Lipophilic Components of GSO

According to published research results, depending on the type and age of the grapes, the quantity of oil in grape seeds ranges from 13 to 15%. The elevated lipophilic content of GSO, which includes vitamin E, unsaturated FAs (UFAs), and phytosterols, has contributed to a rise in interest in the oil [1]. 

#### 5.1.1. Fatty Acids

Nearly 90% of all the FAs in GSO are UFAs. Depending on the type of seed studied, cold-pressing processes generate 20–40% of the monounsaturated FA (MUFA) oleic acid (C18:1n-9) and 65–75% of the PUFAs is LA (C18:2n-6). Around 10% of the total fats are saturated FAs, or SFAs [37]. The FA concentration in grape seeds oil (*V. vinifera* L.), achieved using various extraction techniques, is shown in Table 1. 

Most of the FAs found in GSO, about 90%, are unsaturated. Polyunsaturated acids, particularly LA, are the best represented (65–75%), regardless of the extraction technique utilized. Oleic acid covers up to 20–40% of the monounsaturated acids, depending on the type of seed that was examined. Due to the fact that vegetable oils’ FAs are susceptible to oxidation, GSO’s shelf-life is influenced by its lipid content [37]. It is essential to take this aspect into consideration when the oil is used in food or in the cosmetic and pharmaceutical industry, in order to preserve its properties during the conservation period.

As it cannot be produced by the body, LA, which is present in GSO, has a crucial role and contributes to the substantial nutritional value of foods including it [47]. This is significant since animal studies show that LA boosts cardiovascular health [48]. Furthermore, based on the concentration and oxidative stability of their molecules, these PUFA may alter the GSO’s flavor, aroma, and shelf life [37]. Additionally, LA is a structural component of ceramides and phospholipid cell membranes in the stratum corneum, and it controls trans epidermal fluid retention and the homeostasis of the lipid barrier [2]. Gamma-LA, also an Omega 6 polyunsaturated acid, was detected in GSO in small amounts of 0.1–0.2%. A C18:3 unsaturated Omega-3 fatty acid, α-linolenic acid, is another necessary fatty acid, which in GSO is found in a quantity 10 times smaller than in olive and avocado oil [2]. 

The skin does not have α- or γ-linolenic acids as structural elements. Even so, they contribute to the skin’s biochemical processes of PUFAs along with LA [49]. Skin dysfunctions such as inflammation and dryness have been linked to dietary deficiencies in LA excluding α-linolenic acid [50]. As for the monounsaturated acids in GSO, they are represented by oleic acid, an Omega-9 acid, which is found in a proportion of 14–20%. Most vegetable oils and butters often contain oleic acid, an Omega-9 fatty acid, necessary to human health, poorly represented in grape seeds oil, having a concentration five times lower than in olive oil, the most used oil in skin preparations [2]. It causes permeability irregularities in the stratum corneum structure, which facilitates skin penetration [51,52]. Increased trans-epidermal water loss and irritation are consequences of skin barrier damage [53]. 

The PUFA/SFA ratio is an important indicator of the negative effects exerted by saturated FAs (SFA) on blood cholesterol. At the same time, the recommended ratio of PUFAs to saturated FAs (PUFA/SFA) is preferably greater than 0.4. GSO obtained by different extraction methods fulfills this condition, but the ratio being quite high, between 2–6, shows the fact that this oil requires special storage conditions to prevent oxidation [54].

#### 5.1.2. Vitamin E Isomers

**Figure 5:**
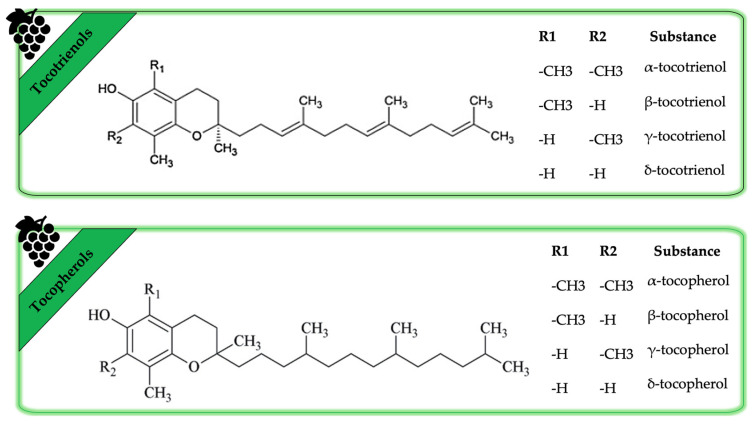
The chemical structure of several tocopherol and tocotrienol types.

Depending on the growth conditions and the type of grape studied, the second category of lipophilic compounds provides up to 50 mg of vitamin E per 100 g of GSO [37]. This class includes eight isoforms of tocopherols and tocotrienols that are liposoluble. Tocopherols and tocotrienols can be quantified by high-performance liquid chromatography. There are four tocopherols (α-, β-, γ- and δ-tocopherol) and four tocotrienols (α-, β-, γ- and δ-tocotrienols). They each function as free-radical scavengers in cell membranes and lipoproteins. Tocopherols play an important role as a quality parameter in the oil by protecting it against lipid oxidation. Furthermore, tocopherols play an important role in the skin as protection against lipid cell membrane oxidation which can lead to inflammation and apoptosis [55]. Tocopherols appear to be universal constituents of all higher plants whereas tocotrienols are thought to be present in a limited number of species and tissues [56]. Figure 5 highlights the structure of the different forms of tocopherols and tocotrienols found in GSO.

**Figure 6:**
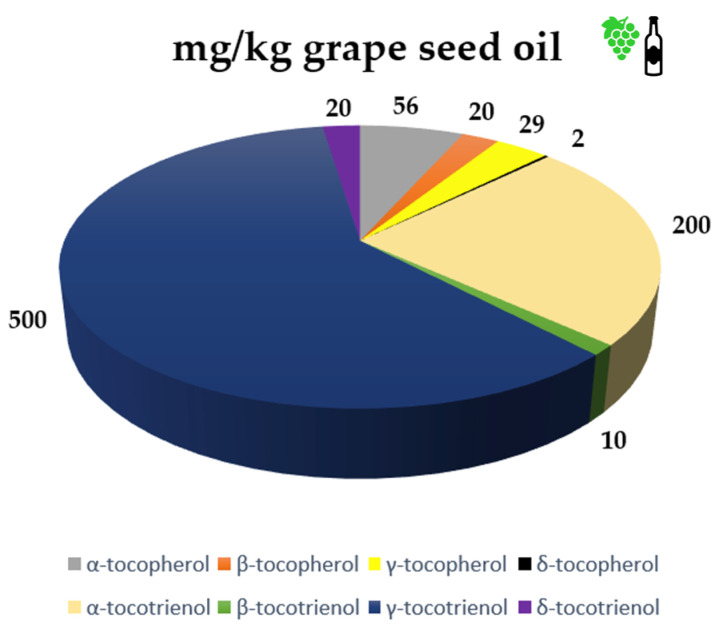
Concentration of tocopherols and tocotrienols in GSO.

According to experimental data, the degree of grape seeds and berries maturity also influences the occurrence of both isomers. The large range of evidence from GSO may be explained by the finding that tocotrienol concentration increases with seed growth and even surpasses that of tocopherols, whilst tocopherol levels reduce during seed maturation [37]. Generally, the tocotrienols content is higher compared with tocopherols. Tocotrienols outnumbered tocopherols by a factor greater than ten in the study conducted by Nikoleta Đorđevski, which examined the oil extracted using Soxhlet method from the kernels of Tamjanika, a native variety of *Vitis vinifera* L. found in the Balkans [45]. The most prevalent tocotrienol was γ-tocotrienol, with a concentration of 23.74 mg/100 g. In 2020 Ustun Argon et al. noted that the forms of α-tocotrienol and γ-tocotrienol represent the most prevalent tocotrienols and that these two elements differ most amongst grape varieties [57]. Figure 6 depicts GSO’s qualitative and quantitative composition (mg/Kg of oil) regarding tocopherols and tocotrienols.

The γ-tocopherol is recognized as a crucial component with antioxidant properties, due to its poor presence in other oils, as well as the fact that mammals cannot synthesize it, thus it must be included in the diet. Compared to white grape seeds, oils from red grape seeds demonstrated a stronger antioxidant capacity, possibly due to the increased amount of α- and γ-tocopherol [1]. GSO is also indicated for older skin because it contains vitamin E, which has antioxidant and anti-aging properties [58]. 

Other kinds of vegetable oils have the highest concentration of α-tocopherol, which is a key component of vitamin E and a dominant antioxidant that scavenges reactive oxygen species (ROS) produced by the body, enhancing the defense mechanism. In membrane phospholipids, α-tocopherol has a role in preventing the degradation of PUFAs [59]. 

A GSO fraction soluble in methanol rendered a mixture α-, γ-tocotrienol, and γ-tocopherol with remarkable anticancer and antioxidant properties and considerable health benefits [60]. 

G. Horvath et al. conducted a remarkable investigation on tocotrienol and tocopherol accumulation in *Vitis vinifera* L. cv. Albert Lavallée (Royal) seeds during seed development. Tocopherols and tocotrienols can both be produced by this species, which is why it was chosen [45,56]. Here, it is demonstrated for the first time that tocopherol and tocotrienol distribution and accumulation kinetics during seed evolution considerably differ from one another. The study’s findings indicated that tocopherol and tocotrienol metabolic activity and retention in growing seeds vary significantly from one another. Since a tocotrienol deposit primarily correlates with oil accumulation during a brief period of the seed formation process, and because tocopherol levels decline with seed age, our findings imply that tocotrienols may play a role in the preservation of stored oil against oxidative stress. While tocopherols are prevalent in the α-form, tocotrienols are usually found in the γ-form. Moreover, tocopherols are widespread in all studied tissues, tocotrienols are only present in endosperm tissue. The presence of tocotrienols was also associated with in vitro biosynthetic activity, especially in the separated endosperm fraction [56]. 

In their study, Veysel U. Celenket al. analyzed tocopherol validation and sample preparation using high-performance liquid chromatography with a fluorescence detection (HPLC-FLD) technique that allows the simultaneous evaluation of 15 distinct cold-pressed oils from Turkey. The following values were found in GSO (µg/mL oil): α-tocopherol 82.37 ± 1.32, β-tocopherol 0.27 ± 0.01, γ-tocopherols 83.84 ± 1.42, and δ-tocopherol 20.24 ± 0.41 [26]. When the oils from the seeds of 21 different grape types (*Vitis* spp.) were examined, it was discovered that α-tocopherol predominated (range from 260.5 to 153.1 mg kg^−1^ oil extract). The overall means of δ-tocopherol, β-tocopherol, and γ-tocopherol, were also found to be 0.92, 0.98, and 22.2 mg kg^−1^ [61]. Seeds of grapes used for Spanish wines having protected denomination of origin (PDO) were used to make the oil that J. C. Bada et al. examined. The predominant tocopherol was α-tocopherol. The tocotrienol found in the largest amounts was α-tocotrienol, which made up 13.18 mg/100 g of oil [62]. 

It is difficult to extract and analyze tocopherol in all its forms since they must be shielded from air and light. Due to the fact of this, sample pretreatment is essential when recording tocopherol values in order to prevent deterioration; direct examination can be carried out after dilution of the oil using an organic solvent prior to quantitative measurement, for instance using HPLC [39]. 

According to Rita de Cássia de Souza et al., supercritical fluid extraction of α-tocopherol with and without ultrasonic pretreatment resulted in up to 14.2-fold greater quantities of α-tocopherol than cold extractions and pretreatment Soxhlet. The increased affinity of alpha-tocopherol to CO_2_ also makes extraction at the start of SFE possible, and tocopherol is then diluted in oil and extracted over the course of the extraction. Tocopherol is selectively collected from the natural matrix in the initial phases of extraction, which is typically obtained following the initial hour of extraction. Due to the competitive separation of other components at a higher temperature using Soxhlet extraction, the extended extraction period and temperature may cause a reduction in vitamin E content. In addition to genotype, environment, climate, growing parameters, location of production, variety, the external conditions during harvest and storing, and obtaining procedures also influence the vegetable oil’s chemical structure and vitamin E content [27]. 

#### 5.1.3. Phytosterols

**Figure 7:**
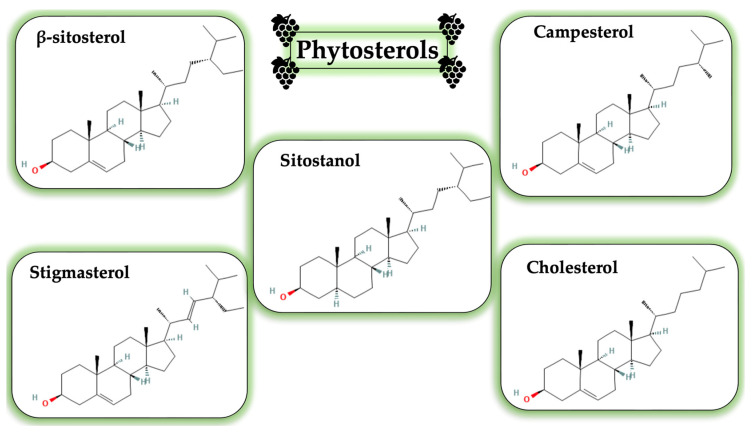
Chemical structure of the most common phytosterols identified GSO.

Phytosterols, which make up about 87–100 mg/kg of GSO, are the third group of lipophilic components [37,39]. Sterol proportion in seed oil is influenced by harvest factors and the method used to extract the oil, as it has already been noted for other constituents. In GSO, β-sitosterol had the greatest proportion (up to 65%), significantly exceeding stigmasterol (the second-highest concentration, about 10%). According to this information, the three sterols that are typically most prevalent in plants are β-sitosterol, campesterol, and ∆5-stigmasterol. Phytosterol’s impact in cholesterol metabolism and antioxidant activity contributes to its biological significance. The cardioprotective action has been demonstrated in vitro, in particular, by β-sitosterol and polyphenols from the wine industry, which inhibits the generation of pro-atherogenic and pro-inflammatory compounds [37,63]. Figure 7 highlights the most common phytosterols found in GSO.

The phytosterols included in GSO may stop oxidized low-density lipoprotein–stimulated macrophages from releasing proinflammatory mediators throughout oxidative stress and eicosanoid production [39].

GSO obtained from red grape seeds from Spain region and analyzed by Jose Emilio Pardo et al., exhibited low cholesterol levels, that were lower than the threshold of 0.5% agreed by guidelines. Stigmasterol, campesterol, clerosterol, and brassicasterol were all found in significant concentrations in the oil from this type of grape seed (dried at ambient temperature). β-sitosterol was the predominant sterol, but the value of this compound was lower in the variety dried at room temperature. The sterol content was influenced by the drying procedure [64]. 

Total phytosterol extraction was higher with Supercritical Fluid Extraction than extraction with solvents, for example, with petroleum ether. In both extracted methods, phytosterols were a prominent feature of the unsaponifiable fraction, with β-sitosterol quantitatively most important with both extractants [65]. 

The by-products of the grape juice extraction that were used to make GSO had a sterol profile that included stigmasterol (10.48%), campesterol (10.64%), β-sitosterol (74.74%), as well as other sterols in smaller amounts. FAs and sterols were analyzed using the gas chromatography (GC) system, using separate columns after oil samples were saponified and methyl-esterified [66]. 

#### 5.1.4. Carotenoids

Carotenoids are part of the category of products with lipophilic antioxidant action. Their presence in GSO depends a lot on the oil extraction method [39]. Thus, Herman Lutterodt et. al, made the carotenoids profile from the defatted flours and cold-pressed GSOs of concord, muscadine, chardonnay, and ruby red grapes. The defatted flour obtained from grape seeds and GSO was mixed with hexanes and subjected to HPLC-MS analysis. According to the findings, only Ruby Red flour had zeaxanthin (157 μg/g of flour), and the greatest levels of β-carotene and cryptoxanthin (2098, 4490, and 50.2 μg/g of flour). The amount of lutein in muscadine flour was the greatest (950 μg/g of flour). Carotenoids were the least abundant in Concord flour. Under the experimental conditions, there was no detectable lutein, β-carotene, cryptoxanthin, or zeaxanthin, in the indicated cold-pressed GSOs [10]. Additionally, from several samples of GSO, obtained by cold pressing, but bought from Brazilian markets, Fernanda Branco Shinagawa et al. obtained a total content of carotenoids, between 33.85–59.85 mg/100 g oil [67]. 

Nikoleta Đorđevski et al. analyzed the content of total carotenoids in GSO obtained by Soxhlet methods with n-hexane. The total carotenoids present in the oil were 0.27 mg/100 g. Just lutein (0.15 mg/100 g oil) was identified and measured among the tested carotenoids. There are very few investigations on the carotenoid levels in GSO [45].

The carotenoid concentration of oils is significant because they give color, but during the extraction process, there is the possibility of significant loss of many of these minor compounds due to their low oil solubility [67]. GSOs contain carotenoids as well, with different quantities, depending on the seed type and extraction technique. Using supercritical CO_2_, it is feasible to obtain more carotenoids than with hexane-based solid-liquid extraction (Soxhlet) [32]. 

### 5.2. The Hydrophilic Compounds of Grape Seeds

#### 5.2.1. Flavonoids and Phenolic Acids

**Figure 8:**
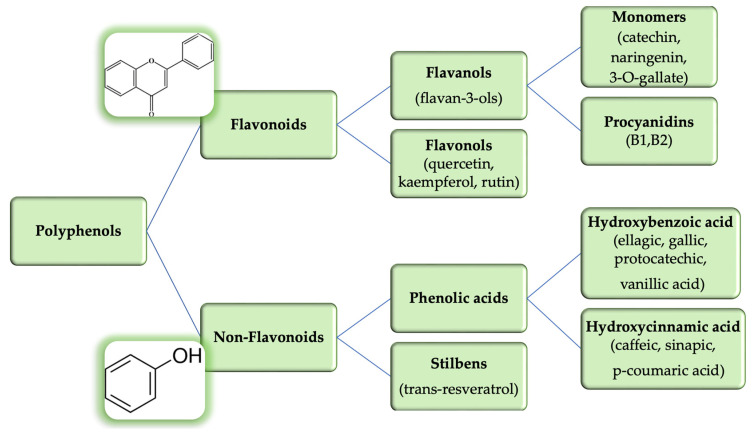
Classification of common polyphenols identified in GSO.

Numerous polyphenolic substances, such as tannins, phenolic acids, stilbenes, and flavonoids, are present in GSO. GSO contains epicatechins, catechins, procyanidin B1, and trans-resveratrol [39]. Figure 8 illustrates the most common polyphenols found in GSO [10,27,68]. 

A grapefruit has three unique tissues (pulp, skin, and seeds), each of them containing a different variety of chemicals, including phenolic acids and polyphenols. The grape berry’s skin contains pigments and tannins, and the seeds also contain tannins [69].

According to prior research, grape seeds incorporate between 60 to 70 percent extractable phenolic chemicals, compared to 10 and 28 to 35 percent in the pulp and skin. Grape seeds have also been shown to contain other chemical classes, typically in tiny amounts, that differ depending on variety or species, such as flavonols, stilbenes, hydrolysable tannins (including ellagitannins and gallotannins), phenolic acids, and organic acids. The polar elements in grape seed extracts, especially the flavan-3-ols, are credited with most of the qualitative features of wine, along with the nutraceutical and biological benefits, hence the current review concentrates on these components [70].

Total phenolic constituents in GSO are low, and extraction protocols can be optimized to increase the yield of antioxidant compounds. Data obtained so far suggest that mechanical pressing extraction is suitable for the recovery of polyphenols from residues, after oil extraction [37]. 

The phenolic part (which is a member of a class of significant biomolecules of vegetable oil), is responsible for the shelf-life and nutritional value of edible oils that have high antioxidant activity in addition to the sensory flavor. Yet, given the low solubility, only little quantities reach the oil [71]. The highest amount of polyphenolic compounds was determined in the dry residue obtained after oil extraction. Just a tiny fraction of the phenolic substances present in plants are carried into the oil during the pressing process (0.01 mg/g), the majority remaining in the press cake. There are around 2000 times more phenolic chemicals in this product. The favorable sensory characteristics of virgin GSO change as it is stored, with many byproducts, such as acetic acid, ethyl acetate, or ethanol, being noticeable. Seed material fragments that are pressed into the oil cause the oil to degrade more quickly [72]. 

Some authors use, as a method of extracting polyphenolic compounds, the repeated liquid-liquid extraction from GSO with a mixture of alcohol and water in different proportions. Furthermore, the hydroalcoholic extract is used to determine the total content of polyphenols, usually with the Folin-Ciocalteu reagent, and for the determination of flavonoids, high-performance liquid chromatography is used [73,74].

Table 2 highlights the correlation between grape varieties, extracting methods, total phenolic content, and their bioactivity. Following extraction, only GSO is the sample that will be examined. 

The pericarp and seeds of grape berries contain polyphenols and phenolic acids, but their distribution in these structures varies greatly. Even if some categories of phenolic acids and polyphenols are strictly regulated by genetics, environmental conditions including soil, climate, vineyard management, and other environmental factors have a significant impact on the final concentration [69]. Approximately 60% of the oil was found in the endosperm and embryo, whereas 90% of the phenolic components were found in the seed coat [82]. 

The polyphenols content also depends on the method of obtaining the oil. Thus, Konuskan et al. determined the total phenol content in GSO (grape seeds of five grape varieties) obtained by two methods (Soxhlet extraction, cold pressed extraction), by using the Folin-Ciocalteu method. The total phenol content was shown to be higher in the oil obtained by Soxhlet methods. If in the oil obtained by pressing the average total phenolic content was 253.044 mg/kg GAE, by the Soxhlet method an average content of 152.398 mg/kg GAE was obtained [44]. 

The influence of the chosen extraction solvent on the quantity of total phenolic compounds from various culinary oils was also demonstrated by Parry et al. [17]. The total content of phenols was determined from the methanolic extract of vegetable oils most often used in food and the following values were obtained: was greatest in groundnut oil (3.09 mg/100 g oil), then coconut oil (1.8 mg/100 g oil), rice bran oil (0.89 mg/100 g oil), mustard oil (0.56 mg/100 g oil), sunflower oil (0.49 mg/100 g oil), and sesame oil (0.33 mg/100 g oil, respectively [83]. Thus, GSO has a much higher total phenol content than most edible vegetable oils. It is also worth noting that the methanolic extract from white or red grape seeds, obtained without prior degreasing, has a higher total phenol content than the methanolic extract obtained from grape pulp and skin. For the white grape seed extract, total phenol values varied from 168.27 mg GAE/100g FW to 260.22 mg GAE/100g FW, for red grape seed extract, the values were from 238.02 mg GAE/100g FW to 326.18 mg GAE/ 100g FW [84]. 

Natacha Rombaut and colleagues investigated three oil extraction techniques and assessed each one’s effectiveness in obtaining an oil that is high in polyphenols. The three extraction techniques are supercritical CO_2_ percolation, screw pressing, and a combination of these two procedures (Gas-Assisted Mechanical Expression: GAME). The most effective method for making GSO in greater quantity is screw pressing; however, the supercritical CO_2_ technique enables a greater co-extraction of polyphenols with the oil. When compared to screw pressing, the GAME technique enables the extraction of more polyphenols, making it a remarkable procedure [23]. The use of supercritical fluid technology results in an increase in the polyphenol level in oil when compared to screw pressing. SFE enables a rise in oil polyphenol concentration of up to 350 ± 50 mg GAE/kg oil. Comparing the two processes, screw pressing (253 ± 13 mg GAE/kg oil) provided lower oil polyphenol concentration [23,32]. 

Despite being regarded as hydrophilic, these phenolic compounds have relatively little solubility in water. Increased TPC in GSO that was extracted using hexane suggests that the phenolic molecules in GSO have higher hydrophobicity than hydrophilicity. If they are not eliminated during oil refinement, they might act as antioxidants to reduce the oxidation of GSO [85]. These substances play a crucial role in the PUFAs’ oxidative stability in the oil [86]. 

#### 5.2.2. Stilbenes

A group of polyphenolic substances known as stilbenes function as phytoalexins and shield berries against biotic and abiotic damage. Due to its positive impact on human health, attention to this class of substances, particularly resveratrol, has grown recently. They consist of an ethane bridge connecting two aromatic rings. Although grape stems, seeds, and leaves also contain stilbenes, grape skins contain the major portion of this compound [69]. 

Rita de Cássia de Souza et al. identified resveratrol. Given that this substance is found in grape seeds, this was anticipated. As a result, it was feasible to identify resveratrol in all tested samples, with the sample prepared after ultrasound pretreatment (30 °C for 30 min) and a cold extraction with chloroform, having the highest content (8.05 ± 0.01 mg/kg). 

The amount of resveratrol (5.6 mg/kg) determined by Burin et al. for the Cabernet Sauvignon grape is lower than the amount discovered through this method [87]. No considerable variations between the Soxhlet and cold chloroform extractions were found, and the ultrasonic pretreatment had no effect on either of them. Although the extraction process may have an impact on the final content, the overall amount of resveratrol detected in Syrah red GSO implies that it may be a grape cultivar with considerable resveratrol content [27].

#### 5.2.3. Volatile Compounds

Fruit scent is among the most significant elements that fascinate consumers, making it significant for the intense market competition. Numerous substances are included in grape volatiles, such as alcohols, monoterpenes, carbonyls, and esters. Varieties of grapes, societal customs, and meteorological or biological conditions all affect the level of these volatile chemicals [88]. If premium raw materials are used for manufacture, virgin GSO has a nice vinous and fruity aroma and also, a raisin-like flavor as opposed to refined GSO, which is neutral in smell and taste. Due to the high moisture level after pressing the grape juices, the raw material is vulnerable to microbiological and enzymatic deterioration, therefore challenges occur [72]. Volatile compounds are formed through fatty acid metabolism, producing acids, alcohols, esters, and ketones. The chemical composition of the volatile compounds from GSO was assessed by GC–MS, and the identified molecules are presented in Table 3.

Presenting a fruity and herbaceous note, the presence of volatile chemicals suggests that seeds were fermented to produce substances such as isoamyl alcohol and hexanol. The samples included ethyl octanoate and ethyl hexanoate esters, hexanal, pentanal, and furfural aldehydes as well as floral and/or fruity aromas. Contrarily, it should be emphasized that several volatile chemicals that add a disagreeable flavor are generated during the oxidation process, as demonstrated by the detection of styrene, 2-heptenal, and isovaleric acid from cold-pressed Brazilian GSO. Additionally, it was shown along the extraction process, that the volatile chemical profile of oils can be significantly influenced by growing practices and the maturation stage [67]. The authors state that the action of enzymes or bacteria, oxidative breakdown of FAs (such as LA), processing conditions, or even storage parameters could all contribute to the development of these flavor components [90]. 

## 6. Biological Activity of GSO

This study concentrated on the biological action of GSOs, which has drawn attention due to the bioactivity of several constituent parts. Thus, it has been suggested that GSO is a useful dietary supplement that could help prevent or treat physiological abnormalities linked to long-term illnesses. However, there are still a lot of unanswered problems regarding dosage response, bioavailability, and secondary effects in people. Yet, the primary physiological functions should be discussed considering the accountable oil components, found in the specialized literature and reported strictly for the grape seeds oil extracted by different methods [37]. Used in cosmetology as a raw material, GSO (INCI: *Vitis vinifera* (Grape) Seed Oil) has calming, softening, antioxidant, and normalizing properties [58,92]. 

GSO is known to have a positive impact on health since it contains fat-soluble vitamins, PUFAs, and antioxidants such as carotenoids, polyphenolic compounds, and tocopherols (anti-inflammatory, antibacterial, and antioxidant action) [58]. 

### 6.1. Traditional Uses

The most relevant evidence for the use of *Vitis vinifera* L. as a plant species with healing properties are the written (e.g., cuneiforms, epigraphs, papyri, manuscripts) or painted (e.g., frescoes-murals in tombs and temples, vase paintings, sculptures, iconography, mosaics, miniatures) archaeological sources.

Grape varieties have been evaluated, and the characteristic colors of the fruits as well as the cultivation and wine-making techniques in Greek or Roman literary texts such as the *Iliad*, the *Odyssey* written by Homer in the 8th century BC, the *Aeneid* written by Virgil in 70–19 BC, Horace’s *Odes* from 65–68 BC. or the *Holy Books* (e.g., the *Bible*, *Four Gospels*, the *Torah*, the *Talmud,* or the *Koran*). In the Old Testament we are told that after the great water flood, the first thing Noah does is to plant a vine *(Vitis vinifera* L.). There are arguments that in ancient China, as early as 7000 BC. a fermented grape drink was used, attested by the residues taken from the inside of some vessels [93,94].

In antiquity, papyrus and ceramic fragments were the most used as writing material, being common in different periods of civilization. The Ebers papyrus, one of the oldest and best-preserved medical documents of Ancient Egypt, written around 1500 BC, then discovered in the Valley of the Kings, contains recipes for various remedies, in some of which *Vitis vinifera* L. is also mentioned [95]. Grapes are mentioned in both ancient European and Asian mythology and in ancient religious writings, being considered essential for good health. Even in the Americas, explorers and missionaries reported that the fruits of *Vitis vinifera* L. were already being used as medicine by Indigenous Peoples before the arrival of explorers or missionaries in those lands [93].

**Figure 9:**
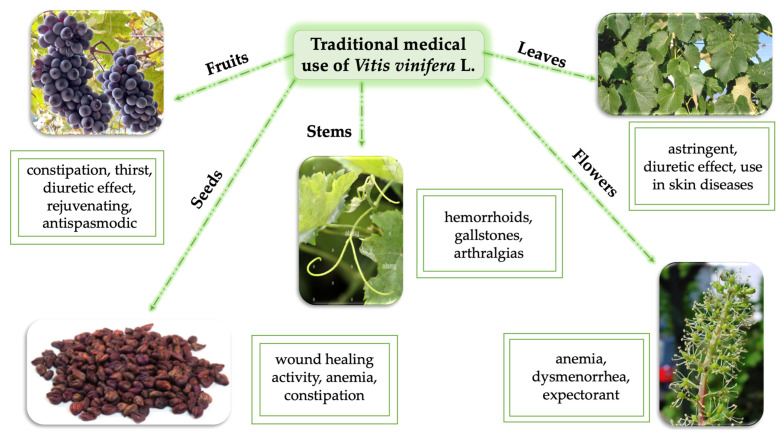
Correlation between several plant parts and their therapeutic actions.

*Vitis vinifera* L. is widely used in the traditional Indian Ayurvedic system of medicine since ancient times for therapeutic purposes, and for the treatment of a variety of ailments [96]. In Ayurvedic recipes, *Vitis vinifera* L. is referred to in various ways, depending on the historical period [97]. Traditional, ancient medicine was plant-based, also using parts of animals and different substances from nature, being a mix between religion and science [98]. In this regard, different parts of *Vitis vinifera* L. were used for their pharmacologic activity as it is presented in Figure 9.

### 6.2. Anti-Hypercholesterolemic and Cardioprotective Effects

High concentrations of PUFAs and phytosterols, which may be beneficial for cardio-metabolic health, can be found in berry seed oils. The main unsaturated fatty acid in grape oil is LA, which has been shown in numerous investigations to support cardiovascular health [39]. 

Pinoresinol, ethyl gallate, and ethyl caffeate, were also found in the cold-pressed GSO and demonstrated an inhibitory action, in type 2 diabetes, against the protein tyrosine phosphatase 1B enzyme (PTP-1B). According to some reports, berry sterols can lower a patient’s cholesterol levels. Due to their antioxidant qualities and influence on cholesterol metabolism, phytosterols, particularly stigmasterol, β-sitosterol, and campesterol in grape oil, are minor lipophilic compounds that present health benefits. Consuming berries helps maintain a healthy gut microbiome and may enhance plasma lipid profiles in humans, thus lowering the incidence of cardiovascular disease [99]. It has been demonstrated that supplementing with GSO considerably lowered triglycerides and LDL-cholesterol values in rats treated with fat diets, which was used to control hyperlipidemia [100].

### 6.3. Antioxidant Potential of GSO

Determining the antioxidant properties of plant materials is among the primary objectives of research efforts since it is related to the high antioxidant content of specific fruits and vegetables with health benefits. Several techniques have been created to assess the antioxidant content in beverages and foods, yet there is currently no agreement on the technique considered the gold standard. This is due to a number of methodological limitations and flaws, including the inability to determine whether an antioxidant is hydrophobic or hydrophilic, the challenges in determining the reaction end point, etc. The antioxidant action of the oil extracted from grape seeds is due to the lipophilic and hydrophilic compounds in its composition [17]. 

The high content of vitamin E isomer, specifically γ-tocotrienol, which is seldom present in other oils, is directly associated with the GSO antioxidant activity, according to evidence in the literature. Studies have shown that tocotrienol-rich fractions have antioxidant and antitumor properties [60,101,102,103]. When iNOS is decreased and COx-2 is inhibited, tocotrienols have a better antioxidant potential than other tocols isomers. The enhancement of insulin sensitivity is one of tocotrienols’ additional significant functions. According to this viewpoint, GSO γ-tocotrienol may account for the oil possible anti-inflammatory and/or antioxidant effects [67]. 

The authors identified different values of the antioxidant capacity of GSO, which could be linked to the various processing methods and variations in the samples’ antioxidant lipophilic and hydrophilic content. Thus, it is confirmed that the method of oil extraction has a significant impact on the antioxidant properties and overall effectiveness of extracts [1]. 

Ivana Dimic, et al., evaluated the antioxidant action of GSO obtained from red and white grape varieties by 2,2-diphenyl-1-picrylhydrazyl (DPPH) and 2,2′-azino-bis(3-ethylbenzothiazoline-6-sulfonic acid) (ABTS) assays. The conclusion of their study was that through Supercritical fluid extraction from red grapes, an oil with the highest antioxidant capacity was obtained [1]. 

### 6.4. Wound Healing Effect

It is believed that the fatty-acid constituents of vegetable oils, particularly PUFAs such as LA, play a significant part in the wound-healing process. Oils with a greater linoleic-to-oleic acid ratio have been found to be more efficient at restoring lipid barriers. To learn more about how vegetable oils affect the skin and vice versa, further in-depth research is required [2]. 

Reducing the negative impacts of free radicals and preserving the structure and stability of biological membranes carotenoids, polyphenols, sterols, and vitamin E, from the GSO composition obtained favorable action on collagen synthesis and wound healing. The humectant action of vitamin E on skin wound scarring was also presented by Palmieri et al. [104]. Sterols are also effective substances that can support lowering systemic inflammation. By activating macrophages and boosting fibroblast and collagen production, they can hasten the formation of new skin [105]. Moreover, the wound-healing action could potentially be linked to a synergy between grape oil containing 20.10 ± 0.02 mg/g hydroxyproline, having wound-healing and antibacterial functions, as demonstrated by the reduction in wound area on the 13th day by 84.6% following grape oil administration. Nayak et al. (2010) revealed that grape skin also demonstrated wound-healing action, the wound area was healed on the 13th day, and antioxidant activity was also reported in the literature [106]. 

### 6.5. Antimicrobial Effect

Many research investigations on grape pomace have concentrated on grape seed extracts (GSE). Gram-positive bacteria respond better to GSE than Gram-negative bacteria, according to prior research. The two-layer cell membrane of Gram-negative bacteria, in contrast to the single-structured membrane of Gram-positive bacteria, is likely responsible for its higher resistance [107]. 

The objective of Nevena M. Dabetic, et al. was to analyze the chemical profile of seed oils isolated from six distinct Serbian white grape types and to establish their efficiency in terms of antioxidant and antibacterial properties. Regarding the antimicrobial action, GSOs obtained by Soxhlet extraction, demonstrated antibacterial action, just against *Staphylococcus aureus*, *E. coli*, *B. subtilis*, *E. faecalis*, and *K. pneumoniae* all grew unaffected by GSO [17]. Even though the major operation mechanism has not been discovered, FAs are considered to have antibacterial action. At low pH levels, they can function as anionic surfactants and exhibit antibacterial and antifungal effects. In order to increase the permeability of the phospholipid bilayers of the membranes, fatty acid hydrocarbon chains can also be integrated into them [17,73]. Polyphenols were also discovered to be efficient on Gram-positive bacteria by attacking cell walls, membrane receptors, lipid membranes, ion channels, biofilm formation, and bacteria metabolites. Other components may also contribute to the antibacterial activity of the examined oils; α-tocopherol has been reported to be more effective against Gram-positive than Gram-negative bacteria [17]. The antibacterial action against *Staphylococcus aureus* (IAL2064) and Escherichia coli (IAL2064) was examined by Tufy Kabbas Junior et al. (ATCC 13565). According to the findings, just the non-polar grape seed and blackberry extracts acquired through Soxhlet extraction showed any inhibitory effects [73].

## 7. Experimental and Clinical Studies

Experimental studies performed in vitro on cell cultures, in vivo on animals, and clinical studies conducted on individuals in order to evaluate the effects of GSO are summarized in Table 4.

## 8. Pharmaceutical Use

### 8.1. Topic Application

GSO is used for pharmaceutical purposes most often, in manufacturing microemulsions and nano emulsions, which are often used as release and transport systems for pharmaceutical substances with different therapeutic effects [116]. 

Microemulsions, through their simplicity and stability, offer the following advantages: spontaneous formation and thermodynamic stability, determining long-term stability and increased homogeneity; are clear/transparent; easy, low-cost preparation, and large-scale transposition is easy; large specific surface of the drops due to their reduced diameter, which implies a large contact surface of the drops with the skin and an increased bioavailability of the medicinal substance; reduced polydispersity of dispersed phase droplets (up to 10%); high capacity to solubilize hydrophilic and lipophilic medicinal substances, due to the high concentration of surfactant; protection of charged molecules in droplets; “reservoir” effect for the medicinal substance [117]. 

Table 5 highlights examples of topic pharmaceutical forms containing GSO. 

### 8.2. Cosmetic Use

The following grape extracts can be used in cosmetics: fruit water, fruit extract, fruit powder, leaf water, leaf oil, leaf extract, leaf wax, leaf/seed skin extract, flower extract, seed extract, seed powder, bud extract, root extract, juice, skin powder, skin extract, vine sap, vine extract, juice extract, and shoot extract [122]. 

The Cosmetic Ingredient Review (CIR) Expert Panel (Panel) formerly examined the safety of grape (Vitis vinifera) seed oil and hydrogenated grapeseed oil in the report Safety Assessment of Plant-Derived Fatty Acid Oils as Used in Cosmetics and came to the conclusion that these compounds are risk-free for cosmetic use [92].

Since it contains FAs, the oil utilized in cosmetics and different personal care items has a favorable effect [123]. The human body is incapable of producing LA; a deficit will cause brittle, dry skin, hair loss, cracked nails, and high trans-epidermal water loss. Due to its ability to effectively treat acne vulgaris, dermatoses, and sunburns, LA is commonly utilized in cosmetic preparations [124]. 

Phenolic compounds and unsaturated fatty acids can be found in high concentrations in GSO. High antioxidant concentrations in grape seeds also have a preventive impact on the skin by boosting cellular resilience and shielding fibroblasts from UV injury by absorbing it. Considering all these factors, grape seeds can bring value to cosmetic compositions. Some sunscreen formulations include extracts with substances that have anti-inflammatory properties to decrease UVB-induced erythema or boost the protection factor (SPF) [125]. 

Grapes constitute a wonderful element to include in cosmetic products because they contain a variety of beneficial compounds. Resveratrol is a great ingredient for cosmetic formulation due to its anti-aging properties and the demonstrated capacity to cross the skin barrier. Additionally, it can promote fibroblast growth and raise the level of collagen III [126]. 

Proanthocyanidins, gallic acid, caffeic acid, ferulic acid, and other phenolic acids and flavonoids are effective antioxidants that may be crucial in the development of post-sun skin care products used in cosmetic surgery [127,128]. Additionally, grapes contain phenolic substances such as catechin, anthocyanins, gallic acid, conjugated flavonoids, epicatechin, oleic, linolenic, and linoleic, acids that counteract epidermal aging manifestations, and slow photoaging progress. SPF and photostability are currently enhanced by the natural antioxidant content of sunscreens. Studies have demonstrated the advantages of applying and ingesting polyphenols from several plant species, including Vitis vinifera, to protect against UV radiation [122]. Table 6 outlines relevant cosmetic varieties containing GSO. 

## 9. Considerations for Manufacturing and Developing GSO Products

Due to its numerous applications, particularly in the therapy against pathogen microorganisms, the production of silver nanoparticles (AgNP) is a very promising option. The antibacterial activity of AgNP is mostly due to their wide surface area, which causes more correspondence involving the cells of the microorganisms and the nanoparticles, inhibiting their growth even at extremely small levels in the medium [139].

There are several studies describing methods of obtaining nanoparticles with the help of active compounds from grape seed extract, especially due to the polyphenolic compounds with antioxidant and antimicrobial action [116]. 

The goal of a study by Niveda Rajeshwaran et al. was to create a gel based on GSO and regulated by silver nanoparticles used for periodontal disorders. They were capable of effectively creating silver nanoparticles and combining them with GSO. Subsequently the properties of the gel formed from the GSO-mediated silver nanoparticles were investigated and it was discovered that the gel possesses antibacterial and nontoxic characteristics [140]. 

The anti-inflammatory and antioxidant properties of GSO gel that have been impregnated with silver nanoparticles were discovered during a further investigation by the same scientists. Silver nanoparticles whose properties and manufacturing are discussed in their prior paper were added to Carbopol, GSO, and water to create the GSO gel. Since grape seed extract is susceptible to numerous modifications during the manufacture of the gel, it is important to determine whether the qualities are maintained. The goal of the study was to evaluate the anti-inflammatory and antioxidant properties of GSO gel infused with silver nanoparticles. According to the findings, GSO gel’s anti-inflammatory and antioxidant characteristics have been preserved, and it is obvious that as the concentration rises, these properties become more active [141]. 

**Figure 10:**
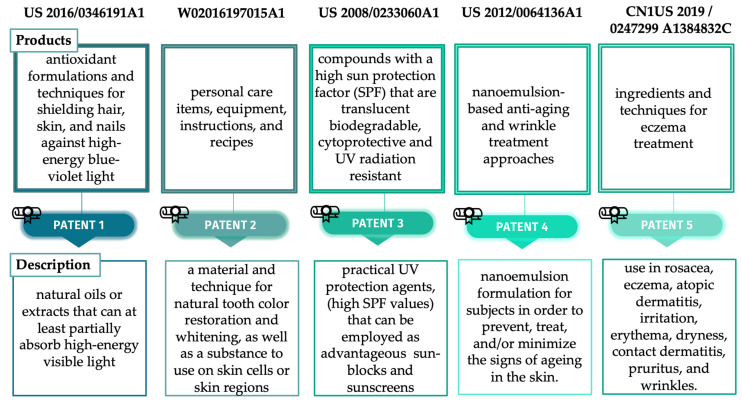
Registered patents with GSO.

Various topical preparations with GSO have been patented, some of the most relevant being depicted in Figure 10.

## 10. Conclusions

This review highlights the multiple benefits of GSO due to its biological compounds, having a potentially beneficial effect in the management of several diseases, and it can also be utilized in the dermato-cosmetics and pharmaceutical industry, being an important source of lipid antioxidants. The extraction methods allow obtaining an oil with a chemical composition that can be exactly determined by modern analysis methods. However, the results reported by researchers differ depending on several factors, and their representation is difficult to achieve due to the way of expression and the different measurement units used by researchers.

Most articles characterize GSO only from the point of view of lipophilic compounds content, namely FAs, tocopherols, and phytosterols. New methods of extracting GSO must be found to increase the quality of the oil by extracting bioactive compounds such as polyphenols, thus increasing its biological activities. These methods must be environmentally friendly, not expensive, and with as little energy and solvent consumption as possible.

## Figures and Tables

**Table 1:** The distribution of fatty acids found in red and white GSO, as evaluated by various extraction techniques.

Grape Variety/Source		Method and Extraction Conditions	Oil Yield % (*w*/*w*)	TOTAL (%)					Refs.
				SFA	MUFA	PUFA	UFA	PUFA/SFA	
Red grapes/ Italy	Sangiovese	Fully automated Soxhlet system n-hexane/isopropanol, 80 °C, 6 h	7.0	7.3	13.2	46	59.2	6.0	[40]
	Montepulciano d’Abruzzo	Decoction in n-hexane, 60% MeOH/H_2_O	10.2	11.6	18.7	69.8	88.5	7.5	[41]
White grapes/ France	Pinot Noir	Soxhlet, Chloroform, 70 °C, 6 h	-	13.0	17.8	69.3	87,1	5	[42,43]
White grapes/ Serbia	Smederevka	Soxhlet Chloroform, 70 °C, 6 h	14.7	14.6	17.5	67.8	85.3	5	[17]
	Tamjanika		17.2	16.1	19.8	63.6	83.4	4	
	Rhine Riesling		15.5	16.0	16.0	66.8	82.9	4	
	Welschriesling		15.7	14.9	17.5	67.1	84.5	4.5	
	Chardonnay		17.4	14.9	18.3	66.0	84.4	4.5	
	Sauvignon Blanc		17.0	15.9	16.1	67.9	84.0	4	
Red grapes/ Turkey	Syrah	Soxhlet n-hexane 80 °C, 6 h	7.7	12.5	21.9	65.3	87.2	5	[44]
		Cold-pressed Bligh and Dyer extraction	-	12.4	21.9	64.9	86.8	5	
	Merlot	Soxhlet n-hexane 80 °C, 6 h	5.6	11.3	17.7	70.8	88.5	6	
		Cold-pressed Bligh and Dyer extraction	-	11.2	21.4	70.0	91.4	6	
	Sangiovese	Soxhlet n-hexane 80 °C, 6 h	4.9	11.1	20.9	67.7	88.7	6	
		Cold-pressed Bligh and Dyer extraction	-	11.0	21.6	67.0	88.7	6	
	Cabernet Sauvignon	Soxhlet n-hexane 80 °C, 6 h	5.6	13.8	20.3	68.1	88.4	5	
		Cold-pressed Bligh and Dyer extraction	-	12.6	20.4	65.8	85.3	5	
White grapes/ Turkey	Sauvignon Blanc	Soxhlet n-hexane 80 °C, 6 h	7.1	13.6	18.7	67.3	86.0	5	[44]
		Cold-pressed Bligh and Dyer extraction	-	13.8	18.5	67.3	85.9	5	
Red grapes/ Serbia	Mixture of Cabernet Sauvignon, Merlot and Pinot noir 65:30:5 (m/m/m)	Supercritical fluid extraction 350 bar, 60 °C	12.2	12.0	13.5	74.4	87.9	6	[1]
		Ultrasound-assisted extraction n-hexane, 40 kHz, 50 °C, 40 min	-	11.2	13.8	74.8	88.7	7	
		Microwave-assisted extraction n-hexane, 600 W, 15 min	-	11.9	16.3	71.7	88.0	6.0	
		Soxhlet n-hexane, 6 h, 15 exchanges of extract	-	11.6	14.1	74.2	88.3	6.5	
White grapes/ Serbia	Mixture of Chardonnay, Sauvignon Blanc, Riesling 60:30:10 (m/m/m)	Supercritical fluid CO_2_ extraction 350 bars, 60 °C	11.8	12.2	17.7	70.0	87.7	6.0	[1]
		Ultrasound-assisted extraction n-hexane, 40 kHz, 50 °C, 40 min	-	12.1	18.5	69.3	87.8	6.0	
		Microwave-assisted extraction n-hexane, 600 W, 15 min	-	11.7	18.0	70.2	88.2	6.0	
		Soxhlet n-hexane, 6 h, 15 exchanges of extract	-	12.1	18.6	69.2	87.8	6.0	
Red grapes/ Brazil	Syrah	Soxhlet n-hexan, 6 h, 60–70 °C	--	34.8	7.3	57.8	65.1	2.0	[27]
		Soxhlet n-hexan, 6 h 60–70 °C ultrasound pretreatment 30 °C, 30 min	-	30.8	6.9	62.1	69.1	2.0	
		Cold extraction Chloroform	-	32.9	6.7	60.3	67.0	2.0	
		Cold extraction Chloroform ultrasound pretreatment 30 °C, 30 min		30.8	6.4	62.7	69.2	2.0	
		Supercritical fluid CO_2_ 50 MPa, 50 °C, 6 g/min, 1.5 h	12.3	31.5	5.6	62.7	68.4	2.0	
		Supercritical fluid CO250 MPa, 50 °C, 6 g/min, 1.5 h ultrasound pretreatment	13.9	31.5	5.6	62.8	68.5	2.0	
White grapes/ Serbia	Tamjanika	Soxhlet n-hexane 60 °C, 6 h	-	11.0	7.4	81.4	88.9	8.0	[45]
Red grapes/ Hungary	Blue Portugal	Cold-pressed Bligh and Dyer extraction	9.5	14.2	16.5	68.8	85.4	6.0	[46]
	Syrah		12.1	13.0	14.2	72.3	85.4	7.0	
	Pinot Noir		13.9	12.3	17.9	69.4	81.7	7.0	
	Cabernet Sauvignon		13.5	13.2	14.4	72.0	85.2	6.0	

SFA, saturated fatty acids; MUFA, monounsaturated fatty acids; PUFA, polyunsaturated fatty acids; UFA, unsaturated fatty acids; MeOH, methanol.

**Table 2:** Total phenolic content and bioactivity of GSO from different varieties.

Grape Variety/Source		Extracting Method	TPC (mg GAE/kg Oil)	Bioactivity	Refs.
White grapes/Hungary	Italian Riesling	Soxhlet-extraction petroleum ether 70 °C, 3 h	1.08	FRAP	[74]
	Cabernet Franc		0.28		
	Királyleányka		1.13		
	Sauvignon Blanc		0.61		
	Rhine Riesling		0.65		
Red grapes/Hungary	Pinot Noir		0.24		
	Merlot		0.97		
	Lemberger		0.28		
Red grapes/Italy	Montepulciano	Extraction n-hexane	12.03	antioxidant properties antimicrobial, anti-inflammatory activity	[41]
	Sangiovese	Soxhlet n-hexane 80 °C 6 h	1.71	DPPH-scavenging capacity	[39,75]
White grapes/Italy	Ribolla Gialla	Soxhlet n-hexane 80 °C 6 h	0.81	DPPH-scavenging capacity	[76]
	Pinot Grigio		1.37		
Red grapes/France	Grenache	Soxhlet, n-hexane 80 °C 6 h Crude extracts were solubilized in water/ethanol followed by Folin-Ciocalteu assay	19.50	Antioxidant Activity (FRAP, DPPH)	[77,78]
	Syrah		24.30		
	Carignan Noir		25.10		
	Mourvèdre		26.30		
	Counoise		20.50		
	Alicante Bouchet		31.60		
White grapes/Chile	Chardonnay	Soxhlet n-hexane 80 °C 6 h	371.50	Antioxidant Activity (FRAP, DPPH)	[79]
Red grapes/Chile	Syrah		327.00		
Red grapes/Argentina	Cabernet Sauvignon	Soxhlet n-hexane 80 °C 6 h	97.57	Antioxidant Activity (FRAP, DPPH)	[80,81]
	Syrah		96.20		
White grapes/China	Chardonnay	Supercritical CO_2_ extraction (28 MPa, 45 °C, 25 kg/h, 75 min)	46.60	DPPH- scavenging capacity	[34]
Red grapes/China	Merlot		80.68		
	Cabernet Sauvignon		98.19		
White grapes/Serbia	Smederevka	Soxhlet extraction chloroform 6 h, 70 °C	104.30	Antioxidant Activity (FRAP, DPPH) Antimicrobial Activity	[17]
	Tamjanika		76.10		
	Rhine Riesling		94.30		
	Welschriesling		91.30		
	Chardonnay		73.40		
	Sauvignon Blanc		100.50		
Red grapes/Turkey	Syrah	Soxhlet n-hexane 80 °C 6 h	182.59	DPPH radical scavenging effect	[44]
		Cold-pressed Bligh and Dyer extraction	148.21		
	Merlot	Soxhlet n-hexane 80 °C 6 h	148.52		
		Cold-pressed Bligh and Dyer extraction	151.51		
	Sangiovese	Soxhlet n-hexane 80 °C 6 h	352.29		
		Cold-pressed Bligh and Dyer extraction	177.30		
	Cabernet Sauvignon	Soxhlet n-hexane 80 °C 6 h	452.99		
		Cold-pressed Bligh and Dyer extraction	182.41		
White grapes/Turkey	Sauvignon Blanc	Soxhlet n-hexane 80 °C 6 h	128.81	DPPH radical scavenging effect	[44]
		Cold-pressed Bligh and Dyer Extraction	102.55		
Red grapes/Serbia	Merlot	Cold pressing	12.66	Evaluation of the free radical scavenging effect on DPPH radicals	[71]
	Hamburg		44.69		
White grapes/Serbia	Italian Riesling		9.29		
	Sila-Serbian autochthonous		11.94		

TPC, total phenolic content; GAE, gallic acid equivalents; DPPH, 2,2-diphenyl-1-picrylhydrazyl, FRAP, ferric reducing antioxidant power.

**Table 3:** Volatile compounds from GSO.

Grape Variety	Identification Methods	Volatile Compounds	Ref.
Solaris grape oil, from the cold-pressed seed of *Vitis vinifera* L.	GC–MS	Hexanal, 1-butanol 3-methyl-acetate, α-pinene, furan 2-pentyl-, hexanoic acid, ethyl ester, d-limonene, octanoic acid ethyl ester	[89]
Virgin grape oils from white and red grapes	HS-SPME coupled to GC–MS	Caproic acid (hexanoic acid), pentanal, hexanal, 2-hexenal, heptanal, trans-2-heptenal and 2-heptanone, 2,3-butandiole, 3- methyl butanol, 2-methylbutanol, hexanol, and ethyl hexanoate, α-pinene, limonene	[90]
Grape seeds oil obtained by mechanical pressing from Syrah, Tintorera varieties and a mixture of Tempranillo, Merlot, and Syrah	SPME and chromatographic analyses	n-octanol, Hexanal, E-2-pentanal, 2-Pentilfurano, Hexan-1-ol, E-2-octenal, Trans-2-hexenal, 1-butanol, 1,3 butanediol, 2,3 butanediol, 1-butanol-3-methylacetato, Heptanal, Pentanol, Styrene, α-pinene, Limonene	[91]

GC–MS, gas chromatography coupled with mass spectroscopy; HS-SPME, headspace solid-phase microextraction, SPME, solid-phase microextraction.

**Table 4:** Experimental and clinical studies regarding the use of GSO.

Main Objective	Conclusion	Ref.
Animal Models		
Rats with excision wounds were used to test the wound-healing properties of cranberry and grape oil	Animals that were administered cranberry and grape oil had considerably more hydroxyproline in their granulation tissue	[108]
Analyze the anti-ulcerogenic and anti-inflammatory properties of *Vitis vinifera* seed extracts (BGSE) and oil (BGSO) in rat experimental colitis	After oral treatment, the hydroalcoholic extract and black grape seed oil displayed protective and prophylactic actions on the acute model of experimental ulcerative colitis, and this effect was highly dosage dependent	[109]
This investigation looked at the impact of GSO on acute liver damage brought on by carbon tetrachloride in rats exposed to γ radiation (7 Gy)	Due to its powerful antioxidant, anti-apoptotic and anti-inflammatory properties, GSO has protective effects on CCl_4_-induced acute liver injury in γ-irradiated rats	[110]
Used an excision wound model to investigate the in vivo wound healing ability of *Vitis vinifera* seed extract with emphasis on wound healing therapeutic targets	In contrast to the Mebo^®^-treated group, the wound healing data showed that V. vinifera seed extract increased wound closure rates, increased VEGF and TGF-β levels, and considerably decreased IL-1β and TNF-α levels	[111]
Cells culture		
By assessing insulin levels and cell apoptosis rates, this study intended to assess the impact of TGSO on elevated glucose-induced Rattus pancreatic β-cell death and identify its signal transduction pathway processes.	The experiment’s findings demonstrated that grape seed oil, which has 87% unsaturated fatty acids, can greatly lower pancreatic β-cell apoptosis and defend pancreatic β-cells	[112]
Individuals		
Compare the effects of a stable water-in-oil (W/O) emulsion with 2% M. Hamburg grape seed extract, to a placebo (the “base”) on human cheek skin	The presented grape-based lotion could be used effectively and safely to treat a variety of skin issues (e.g., hyper-pigmentation, acne, premature ageing)	[113]
To evaluate how olive oil and grape seed oil influence blood pressure and serum lipids in subjects with hyperlipidemia in 2015	Altogether, the benefits of grape seed oil and olive oil were superior to those of the control group. Yet, due to its more advantageous effects, replacing dietary lipids with olive oil is advised	[114]
To assess the impact of foot massage on physiological leg edema in pregnant women when applying sweet almond oil and grape seed oil	This study supported the usefulness of foot massage to alleviate pregnancy-related physiological edema. Sweet almond and GSO were used.	[115]

**Table 5:** Pharmaceutical forms containing GSO with topical application.

Pharmaceutical Form	Ingredients	Use/Effect of GSO	Ref.
Microemulsion	Grape seed oil as oily phase (7.6%), water (23.7%), surfactant–cosurfactant mixture Tween 80 and Plurol^®^ Diisostearique CG (45.2 and 15.1%, respectively), ethanol as the co-solvent (8.4%)	Curcumin’s antioxidant stability is preserved through an improved microemulsion that shields it from external degradants	[118]
Gel microemulsion	Microemulsion GSO and three polymers Carbopol^®^ 980 NF, sodium hyaluronate, and chitosan	Antioxidant	[118]
Nanoemulsifying systems	GSO Cremophor EL, polyethylene glycol 400	Antioxidant activity, and antibacterial activity towards *E. coli*, *B. subtillis,* and *Yeast*	[119]
Nanoemulsifying systems	GSO Nanoparticles α-Tocopherol/casein	Antioxidant	[120]
Nano emulsions	10% oil phase (GSO plus orange oil) 10% surfactant (Tween 80) 80% aqueous phase	Based delivery systems to encapsulate resveratrol	[116]
Nano emulsion	Grape seed oil 19.6%, croduret at 60%, and polyethylene glycol 400 as co-surfactant with a concentration of 16.6%	Increasing the solubility and bioavailability of quercetin.	[121]

**Table 6:** Cosmetical varieties containing GSO.

Name of the Product	Cosmetical Form	Use/Effect of GSO	Ref.
Die Nikolai GSO face cream Nikolaihof Wachau, Austria	Cream	Antioxidants	[129]
Lanolin cream with grape seed (Health care Australia, Chatswood, Australia)	Cream	Linoleic acid from grape seed oil has a considerable amount of antioxidants which are excellent for minimizing signs of skin ageing	[130]
Wine elixir cream with a dense texture (Apivita, Markopoulo Mesogaias, Greece)	Cream	Moisturizes, antiaging effect	[131]
Pigmented cream foundation (100% Pure, San Jose, CA, USA)	Foundation + powder in one	Light and mattifying effect	[132]
Nacomi grape seed oil (Biokera, Wegierska Gorka, Poland)	Oil	Hydration, nutrition, revitalization for the face and neck	[133]
Grape seed oil (Apivita) Markopoulo Mesogaias, Greece	Oil	Hydration, nutrition, softening	[134]
Grape seed scrub (Hillinger Cosmetics, Jois, Austria)	Scrub	Gentle antiaging care for a fresher-looking complexion	[135]
Body wash–vita-rich (Johnson’s, London, UK)	Shower gel	Revitalizing	[136]
Soap (Johnson’s, London, UK)	Solid soap	Cleanse, hydration	[137]
CLBiO Cleansing (CLBiO Co., Ltd. Seocho-gu Seoul, South Korea)	Solid soap	Cleanse, skin protector	[138]

## Data Availability

No new data were created or analyzed in this study. Data sharing is not applicable to this article.

## References

[1] Dimić I., Teslić N., Putnik P., Bursać Kovačević D., Zeković Z., Šojić B., Mrkonjić Ž., Čolović D., Montesano D., Pavlić B. (2020). Innovative and Conventional Valorizations of Grape Seeds from Winery By-Products as Sustainable Source of Lipophilic Antioxidants. Antioxidants.

[2] Poljšak N., Kočevar Glavač N. (2022). Vegetable Butters and Oils as Therapeutically and Cosmetically Active Ingredients for Dermal Use: A Review of Clinical Studies. Front Pharm..

[3] Kremmyda L.S., Tvrzicka E., Stankova B., Zak A. (2011). Fatty acids as biocompounds: Their role in human metabolism, health and disease: A review. Part 2: Fatty acid physiological roles and applications in human health and disease. Biomed. Pap. Med. Fac. Palacky Univ. Olomouc.

[4] Badea M., Di Modugno F., Floroian L., Tit D.M., Restani P., Bungau S., Iovan C., Badea G.E., Aleya L. (2019). Electrochemical strategies for gallic acid detection: Potential for application in clinical, food or environmental analyses. Sci. Total Environ..

[5] Shah M., Murad W., Ur Rehman N., Mubin S., Al-Sabahi J.N., Ahmad M., Zahoor M., Ullah O., Waqas M., Ullah S. (2021). GC-MS Analysis and Biomedical Therapy of Oil from n-Hexane Fraction of Scutellaria edelbergii Rech. f.: In Vitro, In Vivo, and In Silico Approach. Molecules.

[6] Bogdan M.A., Bungau S., Tit D.M., Zaha D.C., Nechifor A.C., Behl T., Chambre D., Lupitu A.I., Copolovici L., Copolovici D.M. (2021). Chemical Profile, Antioxidant Capacity, and Antimicrobial Activity of Essential Oils Extracted from Three Different Varieties (Moldoveanca 4, Vis Magic 10, and Alba 7) of Lavandula angustifolia. Molecules.

[7] Burr G.O., Burr M.M. (1929). A new deficiency disease produced by the rigid exclusion of fat from the diet. J. Biol. Chem..

[8] Burr G.O., Burr M.M. (1930). On the nature and role of the fatty acids essential in nutrition. J. Biol. Chem..

[9] Kaur N., Chugh V., Gupta A.K. (2014). Essential fatty acids as functional components of foods-a review. J. Food Sci. Technol..

[10] Lutterodt H., Slavin M., Whent M., Turner E., Yu L. (2011). Fatty acid composition, oxidative stability, antioxidant and antiproliferative properties of selected cold-pressed grape seed oils and flours. Food Chem..

[11] Jara C.P., Mendes N.F., Prado T.P.d., de Araújo E.P. (2020). Bioactive fatty acids in the resolution of chronic inflammation in skin wounds. Adv. Wound Care.

[12] Sampath H., Ntambi J.M. (2004). Polyunsaturated fatty acid regulation of gene expression. Nutr. Rev..

[13] Ferreri C., Chatgilialoglu C., Ferreri C., Chatgilialoglu C. (2015). Lipidomic Profiles and Intervention Strategies in Prevention and Diseases. Membrane Lipidomics for Personalized Health.

[14] Oprea O.B., Apostol L., Bungau S., Cioca G., Samuel A.D., Badea M., Gaceu L. (2018). Researches on the chemical composition and the rheological properties of wheat and grape epicarp flour mixes. Rev. Chim..

[15] Balić A., Vlašić D., Žužul K., Marinović B., Bukvić Mokos Z. (2020). Omega-3 versus omega-6 polyunsaturated fatty acids in the prevention and treatment of inflammatory skin diseases. Int. J. Mol. Sci..

[16] Csakvari A.C., Andreea L.P., Bungau S., Gitea M., Gitea D., Tit D.M., Copolovici L., Nemeth S., Copolovici D. (2019). Fatty acids profile and antioxidant activity of almond oils obtained from six Romanian varieties. Farmacia.

[17] Dabetic N.M., Todorovic V.M., Djuricic I.D., Antic Stankovic J.A., Basic Z.N., Vujovic D.S., Sobajic S.S. (2020). Grape Seed Oil Characterization: A Novel Approach for Oil Quality Assessment. Eur. J. Lipid Sci. Technol..

[18] Moisa C., Lupitu A., Pop G., Chambre D.R., Copolovici L., Cioca G., Bungau S., Copolovici D.M. (2019). Variation of the chemical composition of Thymus vulgaris essential oils by phenological stages. Rev. Chim..

[19] Sochorova L., Prusova B., Cebova M., Jurikova T., Mlcek J., Adamkova A., Nedomova S., Baron M., Sochor J. (2020). Health effects of grape seed and skin extracts and their influence on biochemical markers. Molecules.

[20] Page M.J., McKenzie J.E., Bossuyt P.M., Boutron I., Hoffmann T.C., Mulrow C.D., Shamseer L., Tetzlaff J.M., Akl E.A., Brennan S.E. (2021). The PRISMA 2020 statement: An updated guideline for reporting systematic reviews. BMJ.

[21] Nassiri-Asl M., Hosseinzadeh H. (2009). Review of the pharmacological effects of *Vitis vinifera* (Grape) and its bioactive compounds. Phytother. Res..

[22] Nowshehri J.A., Bhat Z.A., Shah M.Y. (2016). Pharmacognostic standardisation and phytochemical evaluation on the seeds of two *Vitis vinefera* L. varieties grown in Kashmir Valley, India. Pharmacogn. J..

[23] Rombaut N., Savoire R., Thomasset B., Bélliard T., Castello J., Van Hecke É., Lanoisellé J.-L. (2014). Grape seed oil extraction: Interest of supercritical fluid extraction and gas-assisted mechanical extraction for enhancing polyphenol co-extraction in oil. Comptes Rendus Chim..

[24] Dos Santos Freitas L., de Oliveira J.V., Dariva C., Jacques R.A., Caramão E.B. (2008). Extraction of grape seed oil using compressed carbon dioxide and propane: Extraction yields and characterization of free glycerol compounds. J. Agric. Food Chem..

[25] Rombaut N., Savoire R., Thomasset B., Castello J., Van Hecke E., Lanoisellé J.-L. (2015). Optimization of oil yield and oil total phenolic content during grape seed cold screw pressing. Ind. Crops Prod..

[26] Celenk V.U., Gumus Z.P., Argon Z.U., Buyukhelvacigil M., Karasulu E. (2018). Analysis of chemical compositions of 15 different cold-pressed oils produced in Turkey: A case study of tocopherol and fatty acid analysis. J. Turk. Chem. Soc. Sect. A Chem..

[27] De Souza R.d.C., Machado B.A.S., Barreto G.d.A., Leal I.L., Anjos J.P.d., Umsza-Guez M.A. (2020). Effect of experimental parameters on the extraction of grape seed oil obtained by low pressure and supercritical fluid extraction. Molecules.

[28] Böger B.R., Salviato A., Valezi D.F., Di Mauro E., Georgetti S.R., Kurozawa L.E. (2018). Optimization of ultrasound-assisted extraction of grape-seed oil to enhance process yield and minimize free radical formation. J. Sci. Food Agric..

[29] Li Y., Skouroumounis G.K., Elsey G.M., Taylor D.K. (2011). Microwave-assistance provides very rapid and efficient extraction of grape seed polyphenols. Food Chem..

[30] Hong N., Yaylayan V.A., Vijaya Raghavan G.S., Paré J.R.J., Bélanger J.M.R. (2001). Microwave-assisted Extraction of Phenolic Compounds from Grape Seed. Nat. Prod. Lett..

[31] Cao X., Ito Y. (2003). Supercritical fluid extraction of grape seed oil and subsequent separation of free fatty acids by high-speed counter-current chromatography. J. Chromatogr. A.

[32] Ben Mohamed H., Duba K.S., Fiori L., Abdelgawed H., Tlili I., Tounekti T., Zrig A. (2016). Bioactive compounds and antioxidant activities of different grape (*Vitis vinifera* L.) seed oils extracted by supercritical CO_2_ and organic solvent. LWT.

[33] Jakobović M., Kiš D., Aladić K., Jakobović S., Lončarić M., Jokić S. (2019). Effect of Drying Method on Supercritical CO_2_ Extraction of Grape Seed Oil. Poljoprivreda.

[34] Wen X., Zhu M., Hu R., Zhao J., Chen Z., Li J., Ni Y. (2016). Characterisation of seed oils from different grape cultivars grown in China. J. Food Sci. Technol..

[35] Agostini F., Bertussi R.A., Agostini G., Atti Dos Santos A.C., Rossato M., Vanderlinde R. (2012). Supercritical extraction from vinification residues: Fatty acids, α-tocopherol, and phenolic compounds in the oil seeds from different varieties of grape. Sci. World J..

[36] Ma Z.F., Zhang H. (2017). Phytochemical Constituents, Health Benefits, and Industrial Applications of Grape Seeds: A Mini-Review. Antioxidants.

[37] Martin M.E., Grao-Cruces E., Millan-Linares M.C., Montserrat-de la Paz S. (2020). Grape (*Vitis vinifera* L.) Seed Oil: A Functional Food from the Winemaking Industry. Foods.

[38] El-Beshbishy H.A., Mohamadin A.M., Abdel-Naim A.B. (2009). In Vitro Evaluation of the Antioxidant Activities of Grape Seed (*Vitis vinifera*) Extract, Blackseed (*Nigella sativa*) Extract and Curcumin. J. Taibah Univ. Med. Sci..

[39] Garavaglia J., Markoski M.M., Oliveira A., Marcadenti A. (2016). Grape Seed Oil Compounds: Biological and Chemical Actions for Health. Nutr. Metab. Insights.

[40] Bombai G., Pasini F., Verardo V., Sevindik O., Di Foggia M., Tessarin P., Bregoli A.M., Caboni M.F., Rombolà A.D. (2017). Monitoring of compositional changes during berry ripening in grape seed extracts of cv. Sangiovese (*Vitis vinifera* L.). J. Sci. Food Agric..

[41] Mollica A., Scioli G., Della Valle A., Cichelli A., Novellino E., Bauer M., Kamysz W., Llorent-Martínez E.J., Fernández-de Córdova M.L., Castillo-López R. (2021). Phenolic Analysis and In Vitro Biological Activity of Red Wine, Pomace and Grape Seeds Oil Derived from *Vitis vinifera* L. cv. Montepulciano d’Abruzzo. Antioxidants.

[42] Yunoki K., Tanji M., Murakami Y., Yasui Y., Hirose S., Ohnishi M. (2005). Fatty Acid Compositions of Commercial Red Wines. Biosci. Biotechnol. Biochem..

[43] Crews C., Hough P., Godward J., Brereton P., Lees M., Guiet S., Winkelmann W. (2006). Quantitation of the Main Constituents of Some Authentic Grape-Seed Oils of Different Origin. J. Agric. Food Chem..

[44] Konuskan D.B., Kamiloglu O., Demirkeser O. (2019). Fatty acid composition, total phenolic content and antioxidant activity of grape seed oils obtained by cold-pressed and solvent extraction. Indian J. Pharm. Educ. Res..

[45] Đorđevski N., Stojković D., Živković J., Pljevljakušić D., Ristanović E., Nikolić B., Ćirić A. (2022). Tamjanika, a Balkan native variety of *Vitis vinifera* L.: Chemical characterization, antibacterial, and anti-dermatomycosis potential of seed oil. Food Sci. Nutr..

[46] Szabó É., Marosvölgyi T., Szilágyi G., Kőrösi L., Schmidt J., Csepregi K., Márk L., Bóna Á. (2021). Correlations between Total Antioxidant Capacity, Polyphenol and Fatty Acid Content of Native Grape Seed and Pomace of Four Different Grape Varieties in Hungary. Antioxidants.

[47] Sotiropoulou E., Varelas V., Liouni M., Nerantzis E. (2015). Grape Seed Oil: From A Winery Waste to A Value Added Cosmetic Product—A Review. http://uest.ntua.gr/iwwatv/proceedings/presentations/21_May/SESSION_VI/grape_seed_oil_conference_presentationfinal.pdf.

[48] Kim D.-J., Jeon G., Sung J., Oh S.-K., Hong H.-C., Lee J. (2010). Effect of grape seed oil supplementation on plasma lipid profiles in rats. Food Sci. Biotechnol..

[49] Ziboh V.A., Miller C.C., Cho Y. (2000). Metabolism of polyunsaturated fatty acids by skin epidermal enzymes: Generation of antiinflammatory and antiproliferative metabolites. Am. J. Clin. Nutr..

[50] Ziboh V.A., Miller C.C. (1990). Essential fatty acids and polyunsaturated fatty acids: Significance in cutaneous biology. Annu. Rev. Nutr..

[51] Jiang S.J., Hwang S.M., Choi E.H., Elias P.M., Ahn S.K., Lee S.H. (2000). Structural and functional effects of oleic acid and iontophoresis on hairless mouse stratum corneum. J. Investig. Derm..

[52] Mack Correa M.C., Mao G., Saad P., Flach C.R., Mendelsohn R., Walters R.M. (2014). Molecular interactions of plant oil components with stratum corneum lipids correlate with clinical measures of skin barrier function. Exp. Derm..

[53] Tanojo H., Boelsma E., Junginger H.E., Ponec M., Boddé H.E. (1998). In vivo human skin barrier modulation by topical application of fatty acids. Ski. Pharm. Appl. Ski. Physiol..

[54] Kang M.J., Shin M.S., Park J.N., Lee S.S. (2005). The effects of polyunsaturated:saturated fatty acids ratios and peroxidisability index values of dietary fats on serum lipid profiles and hepatic enzyme activities in rats. Br. J. Nutr..

[55] Blaak J., Staib P. (2022). An updated review on efficacy and benefits of sweet almond, evening primrose and jojoba oils in skin care applications. Int. J. Cosmet. Sci..

[56] Horvath G., Wessjohann L., Bigirimana J., Monica H., Jansen M., Guisez Y., Caubergs R., Horemans N. (2006). Accumulation of tocopherols and tocotrienols during seed development of grape (*Vitis vinifera* L. cv. Albert Lavallée). Plant Physiol. Biochem..

[57] Ustun Argon Z., Celenk V.U., Gumus Z.P. (2020). Cold pressed grape (*Vitis vinifera*) seed oil. Cold Pressed Oils.

[58] Michalak M., Kiełtyka-Dadasiewicz A. (2018). Oils from fruit seeds and their dietetic and cosmetic significance. Herba Pol..

[59] Bouymajane A., Oulad El Majdoub Y., Cacciola F., Russo M., Salafia F., Trozzi A., Rhazi Filali F., Dugo P., Mondello L. (2020). Characterization of Phenolic Compounds, Vitamin E and Fatty Acids from Monovarietal Virgin Olive Oils of “Picholine marocaine” Cultivar. Molecules.

[60] Choi Y., Lee J. (2009). Antioxidant and antiproliferative properties of a tocotrienol-rich fraction from grape seeds. Food Chem..

[61] Sabir A., Unver A., Kara Z. (2012). The fatty acid and tocopherol constituents of the seed oil extracted from 21 grape varieties (*Vitis* spp.). J. Sci. Food Agric..

[62] Bada J., León-Camacho M., Copovi P., Alonso L. (2015). Characterization of grape seed oil from wines with protected denomination of origin (PDO) from Spain. Grasas Aceites.

[63] Behl T., Bungau S., Kumar K., Zengin G., Khan F., Kumar A., Kaur R., Venkatachalam T., Tit D.M., Vesa C.M. (2020). Pleotropic Effects of Polyphenols in Cardiovascular System. Biomed. Pharmacother..

[64] Pardo J.E., Fernández E., Rubio M., Alvarruiz A., Alonso G.L. (2009). Characterization of grape seed oil from different grape varieties (*Vitis vinifera*). Eur. J. Lipid Sci. Technol..

[65] Beveridge T.H.J., Girard B., Kopp T., Drover J.C.G. (2005). Yield and Composition of Grape Seed Oils Extracted by Supercritical Carbon Dioxide and Petroleum Ether:  Varietal Effects. J. Agric. Food Chem..

[66] Alizadeh A., Rofehgarinejad L., Darabi Amin M., Hosseinzadeh Moghbeli A.H. (2016). Fatty acid, phytosterols and tochopherol content of grape seed oil from juicer by-products. Minerva Biotecnol..

[67] Shinagawa F.B., Santana F.C.d., Araujo E., Purgatto E., Mancini-Filho J. (2017). Chemical composition of cold pressed Brazilian grape seed oil. Food Sci. Technol..

[68] Li F.-X., Li F.-H., Yang Y.-X., Yin R., Ming J. (2019). Comparison of phenolic profiles and antioxidant activities in skins and pulps of eleven grape cultivars (*Vitis vinifera* L.). J. Integr. Agric..

[69] Šikuten I., Štambuk P., Andabaka Ž., Tomaz I., Marković Z., Stupić D., Maletić E., Kontić J.K., Preiner D. (2020). Grapevine as a Rich Source of Polyphenolic Compounds. Molecules.

[70] Padilla-González G.F., Grosskopf E., Sadgrove N.J., Simmonds M.S.J. (2022). Chemical Diversity of Flavan-3-Ols in Grape Seeds: Modulating Factors and Quality Requirements. Plants.

[71] Vujasinović V., Bjelica M., Čorbo S., Dimić S., Rabrenović B. (2021). Characterization of the chemical and nutritive quality of cold-pressed grape seed oils produced in the Republic of Serbia from different red and white grape varieties. Grasas Aceites.

[72] Matthäus B. (2008). Virgin grape seed oil: Is it really a nutritional highlight?. Eur. J. Lipid Sci. Technol..

[73] Kabbas T., de Moura C., do Carmo M.A.V., Azevedo L., Esmerino L.A., Tardivo R.C., Kilpeläinen P., Granato D. (2021). Chemical Composition, Antioxidant, Antimicrobial and Cytotoxic/Cytoprotective Activity of Non-Polar Extracts of Grape (*Vitis labrusca* cv. Bordeaux) and Blackberry (*Rubus fruticosus*) Seeds. Molecules.

[74] Kapcsándi V., Hanczné Lakatos E., Sik B., Linka L.Á., Székelyhidi R. (2021). Characterization of fatty acid, antioxidant, and polyphenol content of grape seed oil from different *Vitis vinifera* L. varieties. Oilseeds Fats Crops Lipids.

[75] Canuti V., Frost S., Lerno L., Tanabe C., Zweigenbaum J., Zanoni B., Ebeler S. (2019). Chemical Characteristics of Sangiovese Wines from California and Italy of 2016 Vintage. J. Agric. Food Chem..

[76] Fermo P., Comite V., Sredojević M., Ćirić I., Gašić U., Mutić J., Baošić R., Tešić Ž. (2021). Elemental Analysis and Phenolic Profiles of Selected Italian Wines. Foods.

[77] Ky I., Lorrain B., Kolbas N., Crozier A., Teissedre P.-L. (2014). Wine by-Products: Phenolic Characterization and Antioxidant Activity Evaluation of Grapes and Grape Pomaces from Six Different French Grape Varieties. Molecules.

[78] Rasines-Perea Z., Ky I., Cros G., Crozier A., Teissedre P.-L. (2018). Grape Pomace: Antioxidant Activity, Potential Effect Against Hypertension and Metabolites Characterization after Intake. Diseases.

[79] Mirabal Y., Caramantin Soriano M., Olate-Olave V., Guzmán L., Pyarasani R., John A., Laurie F. (2021). Characterization of five Chilean agribusiness by-products and their potential use as food supplements. Emir. J. Food Agric..

[80] Osorio-Macías D., Vásquez P., Carrasco Villanueva C., Bergenståhl B., Peñarrieta M. (2018). Resveratrol, phenolic antioxidants, and saccharides in South American red wines. Int. J. Wine Res..

[81] Belmiro T.M.C., Pereira C.F., Paim A.P.S. (2017). Red wines from South America: Content of phenolic compounds and chemometric distinction by origin. Microchem. J..

[82] Xu C., Zhang Y., Wang J., Lu J. (2010). Extraction, distribution and characterisation of phenolic compounds and oil in grapeseeds. Food Chem..

[83] Janu C., Kumar D.R.S., Reshma M.V., Jayamurthy P., Sundaresan A., Nisha P. (2014). Comparative Study on the Total Phenolic Content and Radical Scavenging Activity of Common Edible Vegetable Oils. J. Food Biochem..

[84] Yilmaz Y., Göksel Z., Erdoğan S.S., Öztürk A., Atak A., Özer C. (2015). Antioxidant Activity and Phenolic Content of Seed, Skin and Pulp Parts of 22 Grape (*Vitis vinifera* L.) Cultivars (4 Common and 18 Registered or Candidate for Registration). J. Food Process. Preserv..

[85] Yu J., Smith I., Carver J., Holmes B. (2021). Fatty Acid Composition of Grape Seed Oil as Affected by Grape Variety and Extraction Solvent. EC Nutr..

[86] Lin T.-K., Zhong L., Santiago J.L. (2018). Anti-Inflammatory and Skin Barrier Repair Effects of Topical Application of Some Plant Oils. Int. J. Mol. Sci..

[87] Burin V.M., Ferreira-Lima N.E., Panceri C.P., Bordignon-Luiz M.T. (2014). Bioactive compounds and antioxidant activity of *Vitis vinifera* and *Vitis labrusca* grapes: Evaluation of different extraction methods. Microchem. J..

[88] Yalcin H., Kavuncuoglu H., Ekici L., Sagdic O. (2017). Determination of Fatty Acid Composition, Volatile Components, Physico-Chemical and Bioactive Properties of Grape (*Vitis vinifera*) Seed and Seed Oil. J. Food Process. Preserv..

[89] Bocsan I.C., Pop R.M., Sabin O., Sarkandy E., Boarescu P.-M., Roşian Ş.H., Leru P.M., Chedea V.S., Socaci S.A., Buzoianu A.D. (2021). Comparative Protective Effect of *Nigella sativa* Oil and *Vitis vinifera* Seed Oil in an Experimental Model of Isoproterenol-Induced Acute Myocardial Ischemia in Rats. Molecules.

[90] Bail S., Stuebiger G., Krist S., Unterweger H., Buchbauer G. (2008). Characterisation of various grape seed oils by volatile compounds, triacylglycerol composition, total phenols and antioxidant capacity. Food Chem..

[91] Navas P.B. (2009). Chemical composition of the virgin oil obtained by mechanical pressing form several grape seed varieties (*Vitis vinifera* L.) with emphasis on minor constituents. Arch. Lat. Nutr..

[92] Fiume M.M., Bergfeld W.F., Belsito D.V., Hill R.A., Klaassen C.D., Liebler D.C., Marks J.G., Shank R.C., Slaga T.J., Snyder P.W. (2014). Safety assessment of *Vitis vinifera* (grape)-derived ingredients as used in cosmetics. Int. J. Toxicol..

[93] Terral J.F., Tabard E., Bouby L., Ivorra S., Pastor T., Figueiral I., Picq S., Chevance J.B., Jung C., Fabre L. (2010). Evolution and history of grapevine (*Vitis vinifera*) under domestication: New morphometric perspectives to understand seed domestication syndrome and reveal origins of ancient European cultivars. Ann. Bot..

[94] Cappellini E., Gilbert M.T., Geuna F., Fiorentino G., Hall A., Thomas-Oates J., Ashton P.D., Ashford D.A., Arthur P., Campos P.F. (2010). A multidisciplinary study of archaeological grape seeds. Naturwissenschaften.

[95] Harutyunyan M., Malfeito-Ferreira M. (2022). The Rise of Wine among Ancient Civilizations across the Mediterranean Basin. Heritage.

[96] Joshi V.K., Joshi A., Dhiman K.S. (2017). The Ayurvedic Pharmacopoeia of India, development and perspectives. J. Ethnopharmacol..

[97] Nadkarni K.M. (1996). Indian Materia Medica: With Ayurvedic, Unani-Tibbi, Siddha, Allopathic, Homeopathic, Naturopathic & Home Remedies, Appendices & Indexes.

[98] Bungau S., Popa V.-C. (2015). Between Religion and Science: Some Aspects: Concerning Illness and Healing in Antiquity. Transylv. Rev..

[99] Sabra A., Netticadan T., Wijekoon C. (2021). Grape bioactive molecules, and the potential health benefits in reducing the risk of heart diseases. Food Chem. X.

[100] Gorinstein S., Leontowicz H., Leontowicz M., Lojek A., Číž M., Krzeminski R., Zachwieja Z., Jastrzebski Z., Delgado-Licon E., Martin-Belloso O. (2003). Seed oils improve lipid metabolism and increase antioxidant potential in rats fed diets containing cholesterol. Nutr. Res..

[101] Zhang J.S., Zhang S.J., Li Q., Liu Y.H., He N., Zhang J., Zhou P.H., Li M., Guan T., Liu J.R. (2015). Tocotrienol-rich fraction (TRF) suppresses the growth of human colon cancer xenografts in Balb/C nude mice by the Wnt pathway. PLoS ONE.

[102] Lu Y., Zhang Y., Xu D., Wang Y., Pan D., Wang P., Xia J., Yin S., Liao W., Wang S. (2022). Tocotrienol-Rich Fractions Offer Potential to Suppress Pulmonary Fibrosis Progression. Int. J. Mol. Sci..

[103] Magalingam K.B., Ramdas P., Somanath S.D., Selvaduray K.R., Bhuvanendran S., Radhakrishnan A.K. (2022). Tocotrienol-Rich Fraction and Levodopa Regulate Proteins Involved in Parkinson’s Disease-Associated Pathways in Differentiated Neuroblastoma Cells: Insights from Quantitative Proteomic Analysis. Nutrients.

[104] Palmieri B., Gozzi G., Palmieri G. (1995). Vitamin E Added Silicone Gel Sheets for Treatment of Hypertrophic Scars and Keloids. Int. J. Dermatol..

[105] Vitale S., Colanero S., Placidi M., Di Emidio G., Tatone C., Amicarelli F., D’Alessandro A.M. (2022). Phytochemistry and Biological Activity of Medicinal Plants in Wound Healing: An Overview of Current Research. Molecules.

[106] Moalla Rekik D., Ben Khedir S., Ksouda Moalla K., Kammoun N.G., Rebai T., Sahnoun Z. (2016). Evaluation of wound healing properties of grape seed, sesame, and fenugreek oils. Evid. Based Complement. Altern. Med..

[107] Oliveira D.A., Salvador A.A., Smânia A., Smânia E.F.A., Maraschin M., Ferreira S.R.S. (2013). Antimicrobial activity and composition profile of grape (*Vitis vinifera*) pomace extracts obtained by supercritical fluids. J. Biotechnol..

[108] Shivananda Nayak B., Dan Ramdath D., Marshall J.R., Isitor G., Xue S., Shi J. (2011). Wound-healing Properties of the Oils of *Vitis vinifera* and Vaccinium macrocarpon. Phytother. Res..

[109] Niknami E., Sajjadi S.E., Talebi A., Minaiyan M. (2020). Protective Effect of *Vitis vinifera* (Black Grape) Seed Extract and Oil on Acetic Acid-Induced Colitis in Rats. Int. J. Prev. Med..

[110] Ismail A.F.M., Salem A.A.M., Eassawy M.M.T. (2016). Hepatoprotective effect of grape seed oil against carbon tetrachloride induced oxidative stress in liver of γ-irradiated rat. J. Photochem. Photobiol. B Biol..

[111] Al-Warhi T., Zahran E.M., Selim S., Al-Sanea M.M., Ghoneim M.M., Maher S.A., Mostafa Y.A., Alsenani F., Elrehany M.A., Almuhayawi M.S. (2022). Antioxidant and Wound Healing Potential of *Vitis vinifera* Seeds Supported by Phytochemical Characterization and Docking Studies. Antioxidants.

[112] Lai X., Kang X., Zeng L., Li J., Yang Y., Liu D. (2014). The protective effects and genetic pathways of thorn grape seeds oil against high glucose-induced apoptosis in pancreatic β-cells. BMC Complement. Altern. Med..

[113] Sharif A., Akhtar N., Khan M.S., Menaa A., Menaa B., Khan B.A., Menaa F. (2015). Formulation and evaluation on human skin of a water-in-oil emulsion containing Muscat hamburg black grape seed extract. Int. J. Cosmet. Sci..

[114] Kaseb F., Biregani A.N. (2016). Effects of olive oil and grape seed oil on lipid profile and blood pressure in patients with hyperlipidemia: A randomized clinical trial. Food Nutr. Sci..

[115] Navaee M., Rakhshkhorshid M. (2020). Comparing the Effect of Foot Massage with Grape Seed Oil and Sweet Almond Oil on Physiological Leg Edema in Primigravidae: A Randomized Clinical Trial. Evid. Based Complement. Altern. Med..

[116] Davidov-Pardo G., McClements D.J. (2015). Nutraceutical delivery systems: Resveratrol encapsulation in grape seed oil nanoemulsions formed by spontaneous emulsification. Food Chem..

[117] Heuschkel S., Shukla A., Neubert R.H. (2005). Use of microemulsions for topical drug delivery. Percutaneous Absorption.

[118] Scomoroscenco C., Teodorescu M., Burlacu S.G., Gîfu I.C., Mihaescu C.I., Petcu C., Raducan A., Oancea P., Cinteza L.O. (2022). Synergistic Antioxidant Activity and Enhanced Stability of Curcumin Encapsulated in Vegetal Oil-Based Microemulsion and Gel Microemulsions. Antioxidants.

[119] Lu L., Yang P., Chen T., Shen Y., Yao Q., Yan J. (2020). Changes in Biological Activities after Olive Oil, Pomegranate Seed Oil, and Grape Seed Oil were Formulated into Self-Nanoemulsifying Systems. J. Oleo Sci..

[120] Sun L., Wang H., Li X., Lan S., Wang J., Yu D. (2021). Ultrasonic-assisted preparation of α-Tocopherol/casein nanoparticles and application in grape seed oil emulsion. Ultrason. Sonochem..

[121] Pratiwi G., Ramadhiani A.R., Shiyan S. (2022). Understanding the combination of fractional factorial design and chemometrics analysis for screening super-saturable quercetin-self nano emulsifying components. Pharmacia.

[122] Gonçalves S., Gaivão I. (2021). Natural Ingredients Common in the Trás-os-Montes Region (Portugal) for Use in the Cosmetic Industry: A Review about Chemical Composition and Antigenotoxic Properties. Molecules.

[123] Vermaak I., Kamatou G.P.P., Komane-Mofokeng B., Viljoen A.M., Beckett K. (2011). African seed oils of commercial importance—Cosmetic applications. S. Afr. J. Bot..

[124] Afiq M.A., Rahman R.A., Man Y.C., Al-Kahtani H., Mansor T. (2013). Date seed and date seed oil. Int. Food Res. J..

[125] Baroi A.M., Popitiu M., Fierascu I., Sărdărescu I.-D., Fierascu R.C. (2022). Grapevine Wastes: A Rich Source of Antioxidants and Other Biologically Active Compounds. Antioxidants.

[126] Ratz-Łyko A., Arct J. (2019). Resveratrol as an active ingredient for cosmetic and dermatological applications: A review. J. Cosmet. Laser Ther..

[127] Saewan N., Jimtaisong A. (2015). Natural products as photoprotection. J. Cosmet. Dermatol..

[128] Pallag A., Bungau S., Tit D.M., Tünde J., Sirbu V., Honiges A., Horhogea C. (2016). Comparative Study of Polyphenols, Flavonoids and Chlorophylls in *Equisetum arvense* L. Populations. Rev. Chim..

[129] Grape Seed Oil Face Cream^®^, Ecco Verde. https://www.ecco-verde.com/dienikolai/grape-seed-oil-face-care.

[130] Lanolin Cream with Grape Seed^®^, Healthy Care. https://healthycare.com.au/products/lanolin-cream-with-grape-seed-100g.

[131] Apivita Wine Elixir Thick Texture Cream^®^. https://www.drmax.ro/apivita-wine-elixir-crema-rich-50ml?gclid=CjwKCAjw4JWZBhApEiwAtJUN0GldotodTKigWHcZHnRsTos6FnB1rs_D5KeF6Rr1sGrfW7NoXLkakxoCtFYQAvD_BwE.

[132] Fruit Pigmented Cream Foundation, Ecco Verde. https://www.ecco-verde.com/100-pure/fruit-pigmented-cream-foundation.

[133] Face and Body Oil, Nacomi Grape Seed Oil^®^. https://makeup.ro/product/431642/?gclid=CjwKCAjw4JWZBhApEiwAtJUN0L8BnYY18mLGxsRUzFhYDjrUxUyD0BFmhvtazgLJ8Qicj6tXCTq6qhoCwwgQAvD_BwE.

[134] Graoe Seed Oil, The Secret Soap Store Grape Seed Oil 100%^®^. https://makeup.ro/product/568462/?gclid=CjwKCAjw4JWZBhApEiwAtJUN0Ja86A_jS7_iAk_4J7s8CEd7uuf-ezFf9_MwvIW8BBbdJ1yCXvdVIBoCok0QAvD_BwE.

[135] Natural Beauty Products^®^ by Hillinger Cosmetics. https://www.ecco-verde.com/hillinger-cosmetics.

[136] Johnson’s Body Wash—Vita-Rich^®^, Revitalising Grape Seed Oil. https://www.kanbkam.com/ae/en/johnsons-body-wash-vitarich-revitalising-grape-seed-oil-250ml-B07NZ19531.

[137] Johnson’s Body Care^®^, Soap, Grape Seed Oil Nourishing. https://www.kanbkam.com/eg/en/johnsons-soap-grape-seed-oil-nourishing-2724626549146-B098TXWDD6.

[138] Clbio Cleansing Pack^®^—Coconut, Grape Seed, Apricot Kernel, Avocado, Schisandra Chinensis Fruit, Castor Soap. https://dailymed.nlm.nih.gov/dailymed/drugInfo.cfm?setid=3adfd40f-c433-14aa-e054-00144ff8d46c.

[139] Vinicius de Oliveira Brisola Maciel M., da Rosa Almeida A., Machado M.H., Elias W.C., Gonçalves da Rosa C., Teixeira G.L., Noronha C.M., Bertoldi F.C., Nunes M.R., Dutra de Armas R. (2020). Green synthesis, characteristics and antimicrobial activity of silver nanoparticles mediated by essential oils as reducing agents. Biocatal. Agric. Biotechnol..

[140] Rajeshwaran N., Ramamurthy J., Rajeshkumar S. (2020). Green Synthesis of Grape Seed Oil Mediated Silver Nanoparticle and Preparation of Gel-For Periodontal Diseases. Plant Cell Biotechnol. Mol. Biol..

[141] Rajeshwaran N., Ramamurthy J., Rajeshkumar S. (2021). Evaluation of Antioxidant and Anti Inflammatory Activity of Grape Seed Oil Infused With Silver Nano-particles an In Vitro Study. Int. J. Dent. Oral Sci..

